# To cohabit or inoculate? Testing the limits of microbiota transplantation in Manila clams

**DOI:** 10.1093/ismeco/ycag271

**Published:** 2026-09-25

**Authors:** Giovanna Monticelli, Chiara Mascitelli, Giulia Dalla Rovere, Ilaria Bernardini, Nicole Macrì, Maria Elena Martino, Barbara Cardazzo, Maura Benedetti, Alessandro Nardi, Luca Peruzza, Luca Bargelloni, Tomaso Patarnello, Massimo Milan

**Affiliations:** Department of Comparative Biomedicine and Food Science, University of Padova, 35020 Legnaro (PD), Padua, Italy; Department of Comparative Biomedicine and Food Science, University of Padova, 35020 Legnaro (PD), Padua, Italy; Galilean School of Higher Education, University of Padova, 35131 Padua, Italy; Department of Comparative Biomedicine and Food Science, University of Padova, 35020 Legnaro (PD), Padua, Italy; Department of Comparative Biomedicine and Food Science, University of Padova, 35020 Legnaro (PD), Padua, Italy; Department of Comparative Biomedicine and Food Science, University of Padova, 35020 Legnaro (PD), Padua, Italy; Department of Comparative Biomedicine and Food Science, University of Padova, 35020 Legnaro (PD), Padua, Italy; Department of Comparative Biomedicine and Food Science, University of Padova, 35020 Legnaro (PD), Padua, Italy; Department of Life and Environmental Science, Marche Polytechnic University, 60131 Ancona, Italy; Department of Life and Environmental Science, Marche Polytechnic University, 60131 Ancona, Italy; Department of Comparative Biomedicine and Food Science, University of Padova, 35020 Legnaro (PD), Padua, Italy; Department of Comparative Biomedicine and Food Science, University of Padova, 35020 Legnaro (PD), Padua, Italy; NFBC, National Biodiversity Future Center, 90133 Palermo, Italy; Department of Comparative Biomedicine and Food Science, University of Padova, 35020 Legnaro (PD), Padua, Italy; NFBC, National Biodiversity Future Center, 90133 Palermo, Italy; Department of Comparative Biomedicine and Food Science, University of Padova, 35020 Legnaro (PD), Padua, Italy; NFBC, National Biodiversity Future Center, 90133 Palermo, Italy

**Keywords:** prokaryotic community transplantation, microbiota, marine bivalves, water-mediated transmission, community convergence

## Abstract

Microbiota transplantation has emerged as a powerful tool to investigate host–microbe interactions across a wide range of organisms, yet its applicability and ecological limits in marine bivalves remain poorly understood. Here, we experimentally test whether microbiota associated with chronic environmental contamination can be transferred and persist in adult Manila clams (*Ruditapes philippinarum*). We compared two water-mediated approaches: (i) inoculation of a donor tissue homogenate into the water and (ii) cohabitation with live donor clams through shared seawater flow. Donors originated from a chronically contaminated site (Porto Marghera, Italy) while recipients were naïve clams (France), never previously exposed. Each approach was performed with and without antibiotic pretreatment to evaluate whether transient microbiota disruption could enhance donor signal acquisition. Using RNA-based 16S rRNA profiling, we tracked both overall community similarity to the donor prokaryotic community and the presence of donor-exclusive amplicon sequence variants (ASVs) through a Transfer Index. Although similarity-based metrics suggested modest convergence, donor-exclusive ASVs were detected at low levels and did not persist, indicating weak establishment rather than stable colonization. Antibiotic pretreatment did not significantly reduce prokaryotic diversity or total bacterial load, nor did it improve transfer efficiency or promote convergence toward the donor prokaryotic community. Finally, predicted functional profiles further revealed that pathways associated with xenobiotic metabolism in donor clams were not transmitted under short-term experimental conditions. Together, our results demonstrate that prokaryotic community transplantation in adult bivalves is experimentally achievable but results in limited and transient donor community establishment, providing new insight into the host- and environment-driven ecological constraints governing this process.

## Introduction

The microbiome plays a fundamental and increasingly recognized role in shaping host physiology, immunity, and disease outcomes across multiple species. Among microbial communities, the gut microbiome has emerged as a central focus because of its influence on metabolism, immune regulation, and even neurological functions [1–5]. More studies show that altering the gut microbiota influences host responses to health and disease; this has pushed researchers to develop new approaches to test and use these effects experimentally [6, 7]. Thus, microbiota transplantation has become a key approach to explore how microbial community composition influences host physiology. For instance, in mammals, transferring faecal microbiota from humans with obesity into antibiotic (ATB)-pretreated mice yielded measurable shifts in gut microbial taxa and metabolites [8, 9]. Similar approaches have been extended to other organisms, including aquatic species. Research in shrimps showed that gut bacterial transplantation from biofloc system shrimps to water-exchange system enhanced resistance against *Vibrio parahaemolyticus* and promote beneficial taxa [10]. Similarly, defined microbial communities have been shown to improve shrimp growth and immune performance [11]. In fish, experimental manipulation of the gut microbiota through transplantation has been explored across a range of species and experimental settings. For instance, zebrafish has been used as a model organism to investigate the colonization of human-derived gut bacteria and to study host–microbiota interactions [12]. Faecal microbiota transplantation in koi carp has been used to mitigate microbiota disruptions, restoring key bacterial populations, and normalizing critical metabolites such as aromatic amino acids and glutathione-related compounds [13]. Together, these examples show that microbial communities can pass useful functions from one host to another and that host physiology can change when the microbiota is intentionally manipulated.

The microbiota transplantation in bivalves remains largely unexplored, despite the ecological and economic importance of these organisms. Bivalve-associated microbiomes are known to vary across habitats and environmental conditions [14]. Recent studies indicate that when bivalves from different environments cohabitate, they can partially become more similar, mostly because of shared environmental conditions and host identity rather than true microbial transfer [15, 16], while habitat changes can alter microbiota structure [17, 18]. Even though there are still no standard methods for microbiota transplantation in bivalves, recent work has shown that microbial exposures can influence their immune function and disease resistance. In *Crassostrea gigas*, early-life exposure to a microorganism-enriched seawater environment resulted in persistent reshaping of the host microbiota, increased survival upon challenge with Pacific oyster mortality syndrome, and transcriptional reprogramming of immune genes, with correlated changes in DNA methylation patterns [19]. These effects were demonstrated to be visible in the offspring of other species as well, suggesting that microbiota-driven immune conditioning can persist across generations, likely through epigenetic mechanisms [19–22]. Developing methods to manipulate bivalve microbiome could be very important to face increasing environmental challenges, including climate change, pollution, and habitat degradation. However, standardized methodologies for microbiota manipulation and/or transplantation in adult bivalves are lacking, and the stability and functionality of transplanted communities in these hosts are still unknown [23].

Beyond methodological feasibility, the ability to experimentally transfer microbiomes in bivalves would provide a unique framework to test hypotheses about microbiome–mediated stress adaptation. Bivalves inhabit dynamic and often polluted environments, where the microbiome may contribute to host resilience by mediating detoxification processes, modulating responses to a broad range of environmental stressors (e.g. heat waves, hypoxia), and shaping host immune function [24–27]. In this context, a key question is whether donor-associated microbial signatures, and the stress-related functions linked to them, can be transmitted between individuals via seawater, and how robust this transmission is across transfer approaches. Addressing this point is also relevant for developing microbiome-based strategies to improve stress resilience in aquaculture species under increasing pressure from climate change. Here, we compared two strategies: (i) water-mediated inoculation of a tissue homogenized donor microbiota and (ii) microbiota transfer through cohabitation. In addition, we evaluated the influence of ATB pretreatment on the depletion of native recipient microbiota, following the experimental framework proposed by Gallo *et al*. [28]. To achieve this, we selected *Ruditapes philippinarum* clams from two contrasting environments. Donor clams were collected from Porto Marghera (PM; Venice Lagoon, Italy), a highly polluted area where a distinct microbiome enriched in detoxification-related taxa has been described [17, 29]. Recipient clams were naïve individuals obtained from a French aquaculture facility (Satmar), reared under controlled conditions and free from prior contaminant exposure. This donor-recipient design allowed us to compare alternative methodologies for manipulating bivalve microbiota, thereby opening new perspectives for investigating microbiome-mediated adaptation and functional resilience under environmental stress. Rather than modelling microbiota restoration in a dysbiotic host, as often investigated in vertebrate transplantation studies, our approach tested whether donor-associated prokaryotic communities could be transferred to and become established in healthy, non-dysbiotic recipients, thereby assessing the feasibility and ecological limitations of microbiota manipulation in adult bivalves.

## Materials and methods

### Clam collection and maintenance

A total of 600 Manila clams (*R. philippinarum*) were obtained from the Satmar hatchery (France) in November 2024. Upon arrival, clams were acclimated for 7 days in artificial seawater (ASW; 30 PSU, temperature 22 ± 1°C) under constant aeration and fed daily with a commercial algal mix New Coral Fito Concentrate (A.G.P., Italy), a commercial mixture of microalgae composed of *Isochrysis* (T-Iso) (33.3%) + *Nannochloropsis* (31%) + *Tetraselmis* (18%) + *Phaeodactylum* (18%).

In parallel, donor clams were collected from PM lagoon (Venice, Italy; 45°25′29.9″N 12°16′12.8″E). Of these, 60 individuals, randomly split between two replicate tanks (*n* = 30 clams per tank), were maintained as untreated controls (PM), while another 60 (*n* = 30 clams per tank) underwent the same ATB treatment applied to Satmar clams (see below). Both the PM and PM-ATB groups were used solely to characterize the donor prokaryotic communities and were not used for transplantation. The remaining donor clams were allocated to the transplantation trials: one subset was used immediately to prepare the first homogenate inoculum and to stock the donor tanks of the cohabitation systems, while a separate subset, reserved for the second transplantation boost, was maintained for 48 h in large tanks containing water collected from the same PM sampling site before being used for the second homogenate preparation and donor replacement in the cohabitation systems. A schematic overview of the experimental setup is reported in Fig. 1.

**Figure 1:**
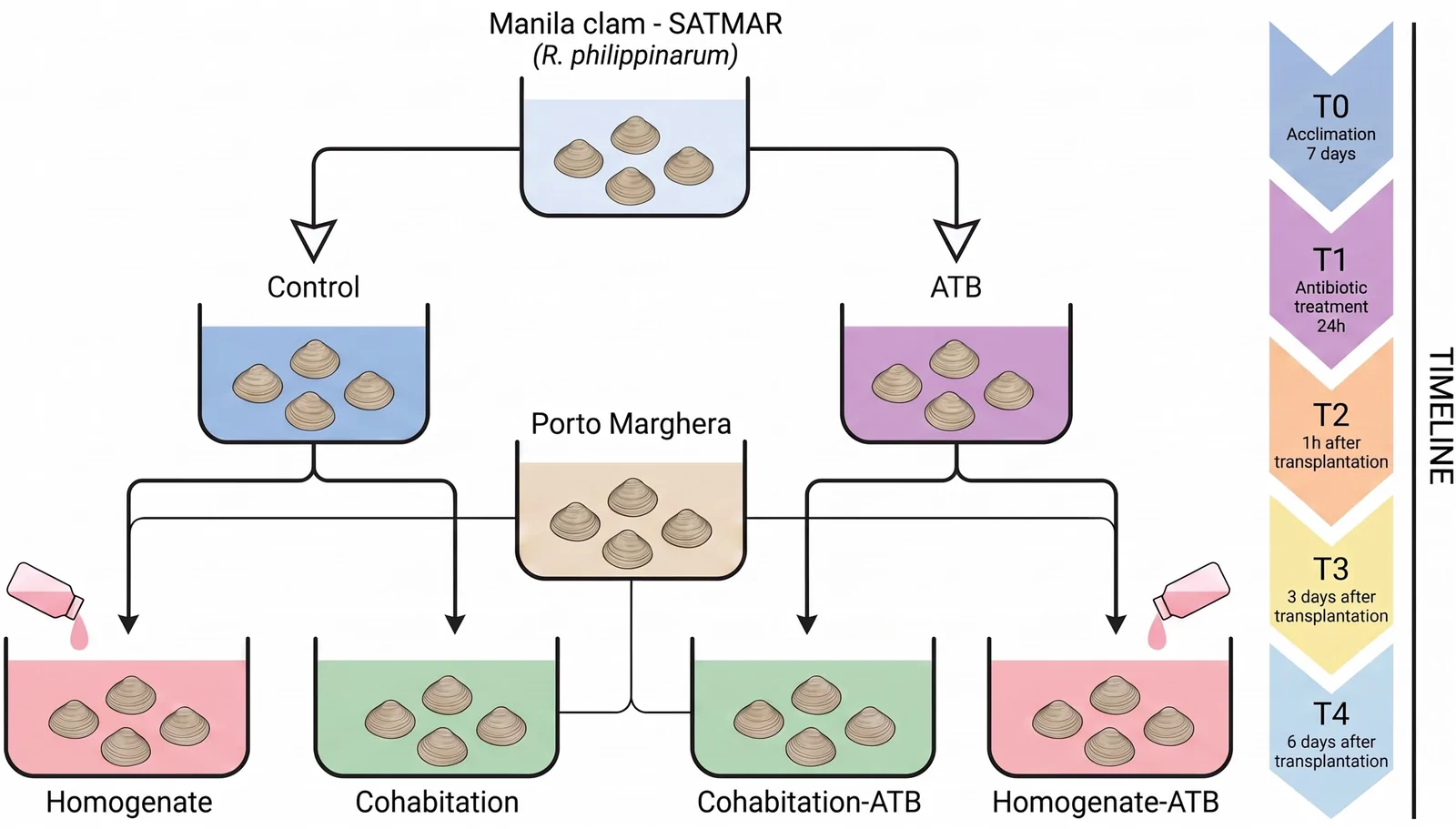
Schematic overview of the experimental design and sampling timeline. Recipient Manila clams (*Ruditapes philippinarum*) from the Satmar hatchery were acclimated for 7 days before baseline sampling (T0) and then divided into two groups: Untreated controls (Control) and antibiotic-treated clams (ATB). Following antibiotic treatment (T1, 24 h), both groups were exposed to donor microbiota from Porto Marghera (PM) clams via two transplantation strategies: Homogenate inoculation or cohabitation, yielding four experimental groups: Homogenate, Cohabitation, Homogenate–ATB, and Cohabitation–ATB. Sampling was performed at five timepoints: T0 (baseline after acclimation), T1 (immediately after antibiotic treatment), T2 (1 h after transplantation), T3 (3 days after transplantation), and T4 (6 days after transplantation, 3 days after the transplantation boost). Tank colours indicate treatment groups throughout the experiment. Created in BioRender. Monticelli, G. (2026) https://BioRender.com/p0f39tb.

### ATB pretreatment

Half of the Satmar recipient clams (*n* = 300) underwent ATB treatment with the aim of reducing their resident prokaryotic communities, following the procedure of Gallo *et al.* [28]. Clams were exposed for 24 h to an ATB mixture consisting of erythromycin (83 mg/l), ampicillin (83 mg/l), streptomycin sulphate (20 mg/l), ciprofloxacin (20 mg/l), and cefotaxime sodium (20 mg/l). The remaining clams (*n* = 300) were maintained in ASW under identical conditions without the ATB mixture and operated as controls. At the end of the 24-h exposure (T1), samples were collected immediately. We then completely replaced the water in both ATB-treated and untreated tanks with fresh ASW and immediately initiated microbiota transplantation.

### Microbiota transplantation methods

Two microbiota transfer strategies were tested using PM clams as donor and Satmar as recipient population:

**Homogenate Transplantation (Homog):** whole soft tissues from 12 donor clams were dissected under sterile conditions, pooled, and homogenized in 5 ml of sterile phosphate-buffered saline (PBS; P4417, Sigma-Aldrich) using a TissueLyser II (Qiagen). The homogenate was sequentially filtered, first through a coarse mesh to remove large debris and then through a 100 μm Corning cell strainer, to obtain a uniform suspension. For transplantation, the homogenate (5 ml) was added to 1 l of sterile ASW containing the recipient clams (*n* = 30 per tank) both ATB-treated and untreated. Clams were incubated in this mixture for 1 h under gentle aeration to allow initial microbial exposure. After 1 h, 5 l of fresh ASW were added to the same tanks, reaching a final volume of 6 l, and clams were maintained under constant aeration for 24 h. A complete water change was then performed to remove any residual homogenate and particulate matter before proceeding with subsequent experimental steps. These steps followed previously published homogenate-based transplantation protocols [28], accounting for the relative number of donor clams and the final ASW volume per tank, with additional methodological details reported in Martino *et al.*. [30].

**Cohabitation transplantation (Cohab):** For the cohabitation approach, a custom two-tank recirculating system was used. The setup consisted of two communicating aquaria (30 l each) connected by a water pump that circulated the same ASW from the lower tank (housing donor clams) to the upper tank (housing recipient clams). This configuration allowed water-mediated exposure of recipient clams to donor-derived microbial communities via shared water flow. Donor clams (*n* = 100 per tank) were placed in the lower tank, while Satmar recipient clams (*n* = 30 per tank) were maintained in the upper tank under constant aeration and identical feeding conditions to the other treatments. ATB-treated and untreated recipient clams were maintained in independent recirculating systems (two independent replicate recirculating systems per treatment), each supplied by its own donor-clam tank. Donor clams used in the different systems were randomly selected from the same PM collection. The number of donor clams and exposure duration were selected to fall within the range of donor densities and renewal strategies previously used in cohabitation studies in oysters [19, 31, 32].

After 3 days, a second transplantation ‘boost’ was performed to enhance microbiota transfer efficiency. In the Cohab treatment, donor clams were removed and replaced with a new set of donors; in the Homog treatment, a freshly prepared homogenate was added to the tanks following the same procedure as described above.

### Experimental design

The recipient design included six groups: untreated control (Control), ATB-treated controls (ATB), homogenate transplantation without ATB (Homog), homogenate transplantation with ATBs (HomogATB), cohabitation without ATB (Cohab), and cohabitation with ATB (CohabATB). Each treatment group was maintained in 2 replicate tanks (*n* = 30 clams per tank). At each sampling timepoint, 3 individuals per tank were sampled (6 individuals per treatment per timepoint), yielding a total of 6 biological replicates per treatment × timepoint combination. In parallel, donor clams were maintained as controls with and without ATB treatment (PM and PM-ATB) in duplicate tanks. Baseline donor sampling (T0) was performed only for the PM group (*n* = 15 individuals).

Sampling was performed at the following time points (Fig. 1):

- T0: baseline (after 7 days of acclimation for Satmar; upon arrival for PM donors).
- T1: ATB treatment (24 h)—end of the 24 h ATB pretreatment for the ATB groups and of the corresponding 24 h maintenance period in ASW for the untreated groups, immediately before microbiota transplantation.
- T2: 1 h after transplantation.
- T3: 3 days after transplantation (pre-boost).
- T4: 6 days after transplantation (3 days post-boost).

### RNA extraction, 16S rRNA sequencing, and analysis

Digestive glands were dissected from six clams per treatment (3 per tank) at each time point, while 15 donor individuals were sampled at T0. Samples were preserved in RNAlater (Thermo Fisher Scientific) and stored at +4°C until processing. Total RNA was extracted individually from each digestive gland using the RNeasy Mini Kit (Qiagen, Hilden, Germany), following the manufacturer’s protocol. RNA concentration and integrity were assessed using a NanoDrop spectrophotometer and Qubit fluorometer.

For RNA-based 16S rRNA profiling, 1 μg of total RNA was reverse-transcribed into cDNA using the SuperScript™ IV First-Strand Synthesis System (Invitrogen™, USA). The V3–V4 hypervariable regions of the bacterial 16S rRNA gene were amplified and sequenced by BMK Gene (Germany) on an Illumina NovaSeq 6000 platform using paired-end 250 bp reads. Raw paired-end reads were imported into QIIME2 (version 2024.5), where primer sequences were removed using the q2-cutadapt plugin (Cutadapt v2.7.8). Denoizing and amplicon sequence variant (ASV) inference were then performed using the q2-dada2 plugin (version 2024.5.0). ASV inference and chimaera detection were performed automatically by DADA2 within QIIME2 (version 2023.9). In total, approximately 1.7% of reads (55 195 out of ~3.2 M) were identified as chimeric and removed at the DADA2 step. Following denoizing, ASVs assigned to mitochondria or chloroplasts at any taxonomic rank, ASVs lacking a Kingdom-level assignment, and ASVs assigned to non-Bacteria/Archaea domains were removed. A prevalence filter was then applied: ASVs detected in only a single sample across the entire dataset were excluded as potentially artefactual (Supplementary Table S1).

Taxonomy was assigned using a Naïve Bayes classifier trained on the SILVA reference database (release 138). Raw sequencing data have been deposited in the NCBI Sequence Read Archive under accession number: PRJNA1362988.

Downstream analyses were conducted in R (version 4.3.2) using the phyloseq package (v1.46.0) for data handling, vegan (v2.7.1) for ecological analyses, and ggplot2 (v3.5.2) for data visualization [33].

Functional profiles were predicted from the ASV table using PICRUSt2 implemented in QIIME2 (q2-picrust2 version 2024.5_1). Predicted pathways were annotated against KEGG orthologues and analysed using STAMP software (v2.1.3) [34–36].

To estimate total bacterial load, the cDNA was analysed by quantitative PCR (qPCR). Total bacterial abundance was quantified by amplifying the 16S rRNA gene using specific bacterial primers (16S_Fw: TCCTACGGGAGGCAGCAGT; 16S_Rev: GGACTACCAGGGTATCTAATCCTGTT), which target the V3–V4 region of the bacterial 16S rRNA gene [37]. All qPCR reactions were performed on a Roche LightCycler 480 Real-Time thermocycler in a total volume of 10 μl, consisting of 2.5 μl of template cDNA (10–50 ng/μl) and 7.5 μl of LightCycler 480 SYBR Green I Master mix (Roche) containing 10 μM of each primer (Eurofins).

For normalization, amplification of 18S rRNA gene from a conserved region among bivalves (bivalve_uni_Fw: CCGATAACGAACGAGACTC; bivalve_uni_Rev: CACAGACCTGTTATTGCTC) was used as reference, allowing relative quantification of total bacterial load in each sample.

### Quantifying microbiota transfer

To quantify the dynamics of microbiome reassembly and the extent of donor signal retention, two complementary metrics were computed:

(i) *Community similarity*

Microbial community structure was analysed using the Bray–Curtis index based on relative abundances (*vegan* R package). For each recipient sample, the Bray–Curtis distance was calculated against the mean relative abundance profile of all PM donor samples at baseline (T0) to reduce inter-individual variability among donor samples.

Temporal trajectories of Bray–Curtis values were then summarized by treatment and timepoint (T2, T3, T4). Because the transplantation phase effectively begins at T2, and the major microbial exchange occurs between T2 and T4, we restricted the temporal analysis to this interval.

(ii) *Transfer Index*

To quantify donor-specific signal, donor-exclusive ASVs were defined as ASVs present in at least one PM donor sample at T0 and absent in Satmar control samples across all timepoints. The donor-exclusive ASV pool was defined once at baseline (PM T0) and kept fixed across all recipient timepoints. For each recipient sample *i* at time *t*:


$$ {\mathrm{TI}}_{i,t}=\frac{\#\left\{\mathrm{donor}-\mathrm{exclusive}\ \mathrm{ASVs}\ \mathrm{detected}\ \mathrm{in}\ i,t\right\}}{\#\left\{\mathrm{donor}-\mathrm{exclusive}\ \mathrm{ASVs}\ \mathrm{in}\ \mathrm{PM}\ \left(\mathrm{T}0\right)\right\}}. $$

Here, ‘#’ indicates the number of distinct ASVs, and TI is computed based on presence/absence rather than relative abundance. ASVs were considered detected when at least one read was observed in the rarefied dataset. Thus, TI measures the fraction of donor-exclusive taxa detected in a recipient relative to the donor-exclusive pool and, by definition, ranges from 0 to 1.

### Functional prediction

Predicted functional profiles were generated from the curated ASV table using PICRUSt2 [38]. Pathways were annotated to KEGG (ko) levels; predicted pathway tables were then converted to relative abundances for downstream statistics, restricted to the same set of samples retained after rarefaction to 3000 reads. To identify donor-enriched functions at baseline, we compared PM donors (T0) versus Satmar Control (T0) in STAMP, applying two-sided tests with Benjamini–Hochberg (BH) false discovery rate (FDR) correction; pathways with FDR < 0.05 were retained as the a priori ‘donor-enriched panel’. For longitudinal evaluation of transplantation outcomes, each treatment at T4 was contrasted against Control (T0) (the baseline reference).

### Statistical analyses

All statistical analyses were performed in R (version 4.3.2). Prior to diversity analyses, the ASV table was rarefied to 3000 retained reads per sample without replacement using the rarefy_even_depth() function implemented in phyloseq (v1.46.0). The rarefaction depth was selected based on joint inspection of sample-level rarefaction curves and the need to preserve adequate replication across treatment × timepoint combinations. Samples with fewer than 3000 retained reads were excluded from diversity analyses. This procedure retained 184 of the 205 samples, while 21 samples were excluded. Sample retention for each treatment × timepoint combination, and sequencing-depth statistics by treatment and by timepoint, are reported in Supplementary Table S2. Sequencing depth was compared separately among treatments and sampling timepoints using Kruskal–Wallis tests. Alpha diversity was estimated from the rarefied dataset using three complementary metrics: observed ASVs, Chao1, and the Shannon index computed with the estimate_richness() function in phyloseq. Pairwise differences in alpha diversity between treatment groups were assessed at each timepoint separately using Wilcoxon rank-sum tests, with BH correction for multiple comparisons (significance threshold: adjusted *P* < .05). Beta diversity was assessed using Bray–Curtis dissimilarity computed on relative abundances. Principal coordinates analysis was performed using the ordinate() function in phyloseq. Permutational multivariate analysis of variance (PERMANOVA; adonis2 in vegan v2.7.1, 999 permutations) was applied separately at each timepoint and as pairwise comparisons at T2, T3, and T4 (BH correction). Homogeneity of multivariate dispersions was verified using betadisper() prior to PERMANOVA interpretation. To explore whether replicate tank identity influenced alpha diversity, Observed ASVs, Chao1, and Shannon indices were compared between the two replicate tanks within each treatment × timepoint combination using Mann–Whitney *U* tests. *P*-values were adjusted using the BH procedure.

To assess the efficacy of ATB treatment in reducing total bacterial load, qPCR was performed on cDNA extracted from the digestive gland of [T0_Satmar control (*n* = 10), T1_Satmar control (*n* = 6), and T1_ATB (*n* = 6)] clams. Pairwise comparisons between groups (T0_Satmar Control vs T1_ATB; T0_Satmar control vs T1_Satmar control) were performed using Wilcoxon rank-sum tests. *P*-values were adjusted for multiple comparisons using the BH procedure.

To identify treatment-associated taxa not evident from multivariate statistics alone, differential abundance analyses were performed with DESeq2 separately for each timepoint, contrasting each transplantation treatment against the corresponding Satmar control group at the same sampling timepoint (T2, T3, and T4). Counts were modelled with treatment group as the explanatory factor, with size factors estimated from geometric means to accommodate ASVs with zero counts and the negative binomial model fitted using a parametric dispersion trend. Pairwise contrasts between each transplantation treatment and the corresponding Control were extracted separately for each timepoint using the contrast argument of the results() function. Taxonomic annotations are reported at the lowest available classification level (genus when available, otherwise higher taxonomic ranks). ASVs were considered significantly differentially abundant at an adjusted *P*-value (BH) < 0.05 and |log2 fold change| > 1.

Donor-specific transfer was evaluated using Bray–Curtis dissimilarity to the donor baseline and the Transfer Index (TI). Temporal trajectories from T2 to T4 were summarized as group means ± SE and visualized descriptively. Statistical testing of treatment effects within Bray–Curtis dissimilarity and TI were performed separately for each time point using linear models or non-parametric tests, depending on data distribution, with FDR correction applied to pairwise comparisons when appropriate. Details of statistical tests and adjusted *P*-values are reported in the corresponding Supplementary Table S3.

Functional pathway abundances predicted by PICRUSt2 were compared using two-sided Wilcoxon rank-sum tests implemented in STAMP, with BH FDR correction (FDR < 0.05). Log2 fold changes were used as effect sizes.

## Results and discussion

Manipulating the microbiota of marine bivalves represents a promising approach to address key ecological and physiological questions, including the role of microbial communities in host resilience to biotic and abiotic stressors.

Our results indicate that microbiota of adult Manila clams is remarkably resilient to external manipulation. Despite using two distinct transplantation approaches, inoculation of donor homogenate, and cohabitation, the shifts observed were only partial and transient.

Sequencing depth (mean ± SD: 9002 ± 3505) did not differ significantly among treatments (Kruskal–Wallis, *P* = .236) or sampling timepoints (*P* = .459), with homogeneous variance across both factors (Brown–Forsythe–Levene tests, *P* = .513 and *P* = .187, respectively; Supplementary Table S2). Sample-level rarefaction curves (Supplementary Fig. S1) confirmed that most samples approached saturation.

### Baseline prokaryotic community characterization

Prior to transplantation, the prokaryotic communities of PM donor clams and Satmar (Control) recipient were compared. At T0, clams differed significantly in composition (PERMANOVA: *F* = 2.49, *R*^2^ = 0.106, *P* = .003), with homogeneous within-group dispersions (betadisper: *P* = .850), confirming a true compositional difference between the two populations. At the taxonomic level, PM donor clams were characterized by higher relative abundances of taxa such as *Endozoicomonas* and *Mycoplasma*, whereas Satmar control clams showed higher relative abundances of *Rhodobacteraceae* and *Vibrionaceae* (Supplementary Figs S2A and Supplementary S3A, D, and G). These differences highlight the distinct ecological signatures of the two populations and provide a baseline to assess donor-derived signal transfer in subsequent transplantation experiments.

### Alpha diversity of prokaryotic communities

We next assessed how diversity changed over time and across treatments (Supplementary Table S4 and Supplementary Fig. S4). Exploratory comparisons of replicate tanks were possible within treatment × timepoint groups (minimum raw *P* = .100; all adjusted *P* = 1.000; Supplementary Table S5); given the small sample size per tank (*n* = 3), these tests had limited power to detect a true tank effect and revealed no consistent tank-associated pattern across comparisons. Both tanks were subsequently pooled for downstream analyses, consistent with the original experimental design.

ATB treatment (T1) did not significantly affect alpha diversity in either PM or Satmar clams (all p_BH > 0.05). Consistent with these patterns, qPCR quantification of total bacterial load revealed no significant differences among T0 control, T1 control, and T1 ATB groups (all p_BH > 0.05; Supplementary Fig. S5). Pairwise comparisons confirmed no significant difference between T0 control and T1 ATB (p_BH = 0.713), nor between T0 control and T1 control (p_BH = 0.957), indicating that the resident microbiota did not show evidence of a substantial reduction. Such incomplete reduction of the resident microbiota may have limited the establishment of donor-associated taxa and contributed to the weak microbiome transfer observed across all transplantation groups.

Following transplantation (T2), Homog clams showed a significant increase in richness, as indicated by both Observed ASVs and Chao1, relative to Control (pBH = 0.025) (Supplementary Table S4; Supplementary Fig. S4C). In contrast, cohabitation treatments showed non-significant effects at this early timepoint. These differences were no longer statistically significant at T3 or T4 after correction for multiple testing (Supplementary Table S4 and Supplementary Fig. S4D, E). Overall, these results indicate that homogenate inoculation is associated with a transient increase in prokaryotic richness, while cohabitation showed no significant effect on alpha diversity.

### Donor-associated microbiota similarity across transplantation strategies

To assess whether recipient communities shifted toward the donor (PM) microbiome composition after transplantation, we included both a fixed PM baseline reference (PM T0) and a contemporaneous PM sample in the Bray–Curtis PCoA (Fig. 2). Communities were significantly dissimilar across groups at all timepoints (global PERMANOVA: all *P* ≤ .003; Supplementary Table S6).

**Figure 2:**
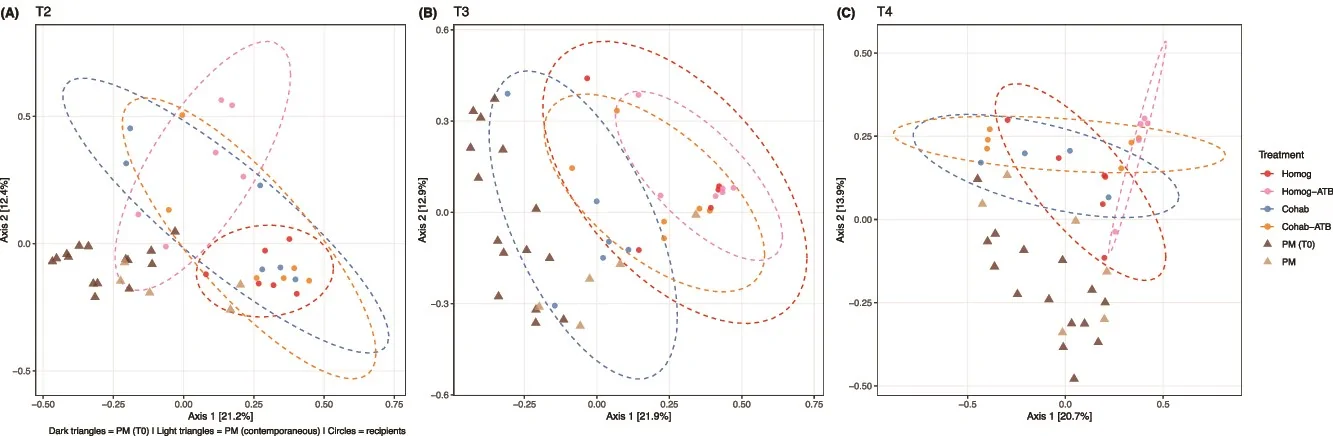
Principal coordinates analysis (PCoA) of Bray–Curtis dissimilarity of prokaryotic community profiles across transplantation treatments at (A) T2, (B) T3, and (C) T4. Each panel includes recipient clams (circles: Homog, Homog–ATB, Cohab, Cohab–ATB), PM donor samples at baseline (PM (T0), dark triangles), and contemporaneous PM donor samples at the corresponding timepoint (PM, light triangles). Dashed ellipses represent 80% confidence intervals for each recipient treatment group. Colours indicate treatment groups as shown in the legend. The percentage of variance explained by each axis is reported in parentheses.

All recipient groups remained significantly distinct from PM T0 at every timepoint (pairwise PERMANOVA, all p_BH ≤ 0.023), showing that recipients did not converge to the original donor baseline at any point during the experiment. Comparing recipients to the contemporaneous PM revealed a more nuanced pattern. At T2, Homog and Homog–ATB were already significantly separated from PM (p_BH = 0.014 for both), whereas Cohab and Cohab–ATB were not (p_BH = 0.128 for both). At T3, all four groups were significantly separated from the contemporaneous PM community (all p_BH ≤ 0.025). At T4, however, neither Cohab nor Cohab–ATB differed significantly from the contemporaneous PM community (p_BH = 0.097 and 0.089, respectively), while Homog and Homog–ATB remained significantly distinct (p_BH = 0.014 for both). Because dispersion was homogeneous at T4 (betadisper, *P* = .839), these differences are unlikely to be driven by unequal variance among groups. However, PERMANOVA significance does not directly reflect compositional proximity, and these results do not provide evidence for convergence toward the donor community or increased incorporation of donor-associated taxa, as also indicated by the TI discussed below. We also note that the PM community itself was not static: it differed significantly from its T0 baseline at T2 (p_BH = 0.020) and T3 (p_BH = 0.009), before returning to a composition statistically similar to T0 by T4 (p_BH = 0.381). This temporal variability in the donor microbiome should therefore be considered when interpreting recipient-donor dissimilarity across timepoints.

Consistent with ordination patterns, Bray–Curtis dissimilarity to the PM T0 baseline remained high across all treatments throughout the transplantation experiment (Fig. 3A), showing no clear decrease over time and indicating limited convergence toward the donor community (Supplementary Table S3). Differences among treatments were generally small at T2 and T3, whereas at T4 Bray–Curtis dissimilarity differed significantly among treatments (Kruskal–Wallis, *P* = .013). Post-hoc comparisons showed that Homog–ATB remained significantly more dissimilar to the PM donor community than Homog, Cohab, and Cohab–ATB (all pBH < 0.05), indicating that ATB pretreatment did not promote convergence toward the donor community. Similarity-based metrics such as Bray–Curtis do not distinguish between donor taxa incorporation and general restructuring of recipient communities.

**Figure 3:**
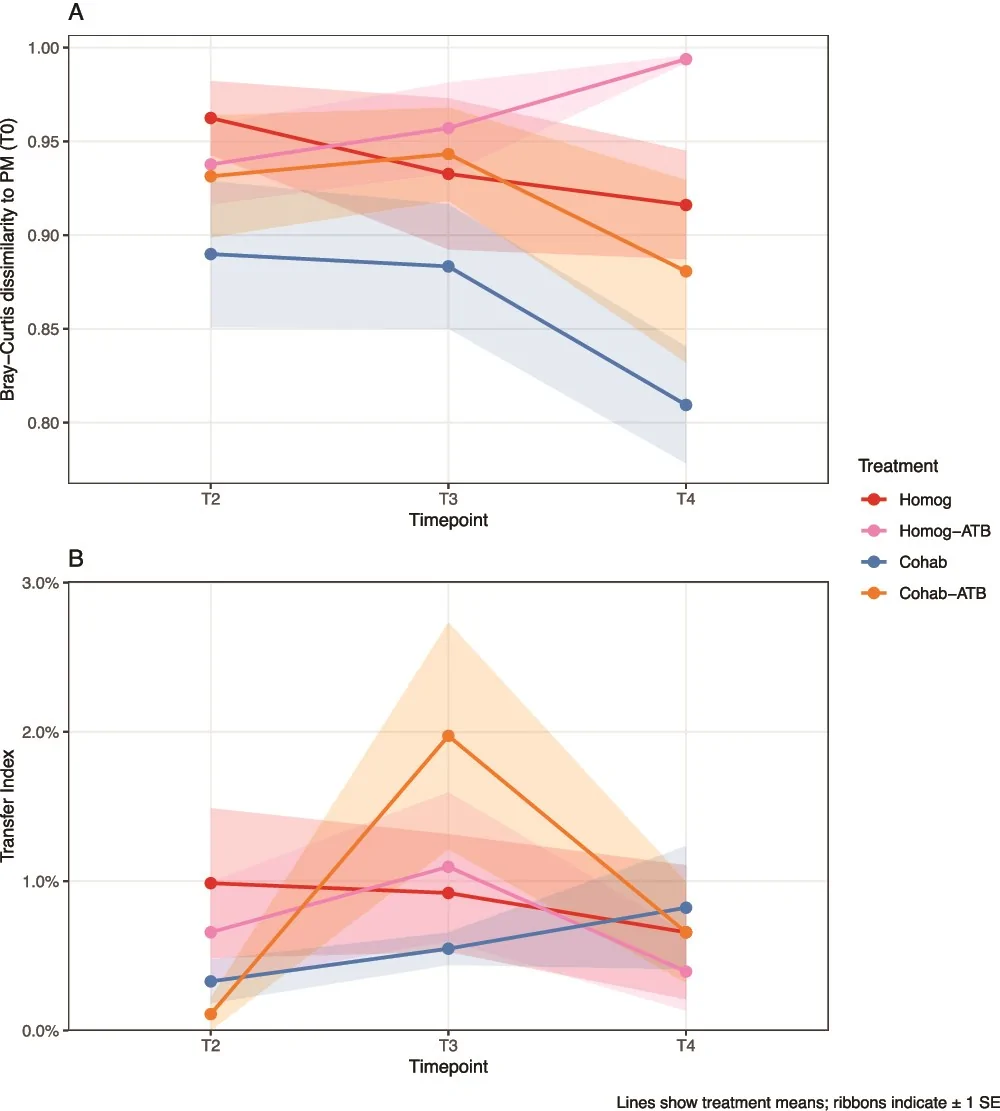
Trajectories of Bray–Curtis dissimilarity to the PM donor baseline and transfer index over time. (A) Bray–Curtis dissimilarity between each recipient treatment group and the PM donor community at baseline (T0), calculated against the mean PM T0 profile. Values approaching 1 indicate complete compositional divergence from the donor baseline. (B) Transfer Index (TI), defined as the proportion of donor-exclusive ASVs, present in at least one PM donor sample at T0 and absent from Satmar Control samples across all timepoints, detected in recipient samples at each timepoint. TI values are expressed as percentages. In both panels, lines represent treatment group means and shaded ribbons indicate ±1 SE. Timepoints T2, T3, and T4 correspond to 1 h, 3 days, and 6 days after transplantation, respectively.

To address this limitation, we used the TI, which tracks the ASVs that are present in donor samples at T0 and absent from recipient controls across all sampling timepoints. TI values remained consistently low across all treatments, indicating that only a small proportion of donor-exclusive ASVs were detected in recipient communities. No significant differences among treatments were detected at any sampling timepoint (Kruskal–Wallis tests, all *P* > .05; Fig. 3B; Supplementary Table S3), indicating that none of the transplantation approaches resulted in greater donor-specific ASV detection than the others. While Bray–Curtis dissimilarity captured changes in overall community structure, TI patterns indicated limited detection of donor-associated ASVs in recipient communities. These results highlight the importance of combining similarity-based and donor-specific metrics as increased similarity to the donor community does not necessarily reflect the acquisition of donor-associated taxa [39–41].

Overall, our findings are consistent with the bivalve digestive gland representing a selectively structured environment in which host-associated ecological filtering may limit colonization by exogenous taxa [42, 43]. The persistence of high Bray–Curtis dissimilarity to the PM donor community, together with low TI values across all treatments, suggests limited integration of the PM donor community, potentially because of the persistence of the native prokaryotic communities and/or host-associated selective pressures. These findings provide no evidence of preferential establishment of specific donor-associated ASVs and are more consistent with general restructuring of recipient communities under shared experimental conditions than with successful donor-specific transplantation.

ATB pretreatment did not enhance donor-specific ASV acquisition, as no significant differences in TI were detected among treatments at any sampling timepoint. Consistently, Bray–Curtis dissimilarity showed that ATB pretreatment did not promote convergence toward the donor community, with Homog–ATB remaining significantly more dissimilar to the donor community than Homog, Cohab, and Cohab–ATB at T4. Since ATB treatment did not significantly reduce resident bacterial load or alpha diversity, these findings suggest that the resident prokaryotic community remained sufficiently resilient to constrain stable establishment of donor-associated taxa despite the transient perturbation induced by ATB treatment. At the same time, prolonged water-mediated exposure may contribute more to overall community convergence than depletion of the resident microbiota alone.

This distinction between global similarity and the uptake of donor-exclusive ASVs is important for interpreting the outcome of the experiment. Comparable patterns have been observed in cohabitating bivalves, where Pacific oysters and Mediterranean mussels shared over 25% of operational taxonomic units (OTUs) just sharing the same ecological niches [15]. Likewise, in mammalian models, such as mice, only a portion of donor ASVs, about 42% of the donor sequence variants, were detected in the recipient [44]. These results support the idea that establishment is driven mainly by ecological convergence rather than by the inoculum or its later boost. Similar dynamics were observed by Ruiz *et al*. in which intestinal microbiome from Atlantic salmon was transferred to gilthead seabream following ATB pretreatment [45]: a few donor-derived taxa established in the recipient over time, with most of the microbiota returning to a profile closer to the native community.

### Taxonomic shifts and differential abundance in recipient communities following transplantation

To identify which taxa drove the observed community shifts, we examined phylum-, family- and genus-level compositional profiles across treatments and timepoints (Supplementary Fig. S3) and performed DESeq2 differential abundance analysis at the ASV level, contrasting each transplantation treatment against the untreated Control at T2, T3, and T4 (Supplementary Fig. S2 and Supplementary Table S7). Taxonomic annotations are reported at the lowest available classification level, as DESeq2 operates on individual ASVs whose taxonomic resolution varies depending on database coverage.

Compositional barplots (Supplementary Fig. S3) show that recipient communities underwent treatment-associated shifts in taxonomic composition following transplantation, with distinct profiles across treatments but no clear convergence toward the PM T0 profile. The PM-associated family *Mycoplasmataceae*, which represents a substantial fraction of the PM T0 microbiota, remained at low relative abundance across all transplantation groups and timepoints, with only minor increases observed in cohabitation and homogenate treatments (Supplementary Fig. S3).

DESeq2 analysis revealed subtle differences in the timing and extent of community restructuring between transplantation strategies (Supplementary Fig. S2). Across timepoints, multiple taxa were differentially abundant relative to controls, indicating that both homogenate and cohabitation approaches induced measurable perturbations of the recipient microbiota. However, the majority of enriched taxa, particularly *Gammaproteobacteria*, including *Amphritea, Nitrincolaceae, Marinobacterium, Oceanospirillum*, were not characteristic of PM donor communities (Supplementary Fig. S2A) and likely represent opportunistic members of resident microbiota responding to experimental disturbance, particularly in response to homogenate-derived inputs. At T2 (1h post-transplantation), *Nitrincolaceae* was already elevated in Homog, Homog–ATB, and Cohab–ATB, while *Oceanospirillum* was enriched in Homog and Cohab, together spanning both transplantation routes regardless of ATB pretreatment. *Endozoicomonas* and *Amphritea* followed a narrower path, appearing exclusively in the homogenate-based treatments. This rapid, broad response, occurring within 1 h of transplantation and independent of ATB pretreatment, could be related to an opportunistic bloom triggered by organic matter input rather than donor-derived colonization [46]. By T3, this early signal had largely faded, while *Vibrio* showed a divergent trajectory between transplantation methods: transiently enriched in homogenate-treated groups at T2 but significantly depleted in Cohab from T3 through T4. Across treatments and timepoints, differential abundance patterns were broadly comparable and did not indicate a consistent or progressive incorporation of donor-associated taxa.

Taken together, compositional and differential abundance analyses indicate that prokaryotic community transplantation altered recipient community structure but did not stably reconstruct the donor microbiota. In this study, while both strategies were water-mediated, homogenate inoculation represented an experimental scenario of acute exposure to donor-derived material, whereas cohabitation was designed to simulate a more gradual, water-mediated microbial exposure process. The fact that neither approach resulted in sustained reconstruction of the donor community over the experimental period is consistent with a potential role of the ecological filtering discussed above [45].

Understanding these limits is essential for designing future transplantation approaches. Our results indicate that long-term modification may require strategies that more effectively address these ecological constraints, such as longer exposure periods or more intensive and optimized repeated administrations beyond the two transplantation events used here. Furthermore, it is also important to consider that RNA-based profiling captures transcriptionally active taxa rather than stable colonizers, which may partly account for the limited detection of donor-exclusive ASVs. Differences in the nature of the donor inoculum, where homogenate treatments introduce a whole-body microbial community, while cohabitation relies on water-mediated and ecologically filtered transfer, may further restrict the persistence of donor-associated taxa within the recipient digestive gland.

### Exploring xenobiotic predicted functions in transplanted microbiota

To explore whether any functional traits from the donor population could be passed to the recipients, we used PICRUSt2 [34, 35] to generate predicted metabolic pathways from the 16S data. This step is important since clams from PM live in a chronically contaminated environment, and their microbiota reflects that history, showing predicted enrichment of pathways linked to xenobiotic degradation, which may potentially be relevant to host responses in chemically impacted environments [17, 29]. On the other side, the recipient clams, from the aquaculture facility Satmar, live in controlled clean conditions and are not expected to show these functions.

Starting from the initial phase, the T0, PCA of predicted functions (Supplementary Fig. S6) showed a separation between the PM from controls at T0 (PC1 ≈ 63%, PC2 ≈ 13%). Consistently, differential pathways analysis identified 28 pathways significantly enriched in PM donors rather than in Satmar controls (*q* < 0.05) (Supplementary Table S8). Among the 28 pathways, there were three that belonged to xenobiotic biodegradation and metabolism: dioxin degradation, chloroalkane/chloroalkene degradation, and naphthalene degradation (*q* < 0.05). At the same time, the naïve aquaculture clams used as recipients lacked these detoxification-associated traits, offering a clear framework to evaluate whether functional qualities can be transiently transferred across hosts through microbial exchange.

To further explore whether functional predictions associated with xenobiotic metabolism were transferred through microbial transplantation, we evaluated the predicted KEGG pathways that were enriched in PM donors at T0 in the transplanted groups at the end of the transplantation (T4). Across the three xenobiotic-related pathways listed above, none showed significant enrichment in any transplantation treatment (all FDR > 0.05) (Supplementary Table S8). Naphthalene degradation was instead significantly depleted relative to Control in Cohab (FDR = 0.012) and Homog–ATB (FDR = 0.005), a shift away from, rather than toward, the PM profile; all other treatment–pathway combinations were non-significant (FDR > 0.05). The observed log2FC ranged from approximately −0.18 to +0.41 depending on the pathway and treatment group. These weak and inconsistent log2FC values indicate that the predicted functional signatures characteristic of the chemically impacted PM population were not transferred to recipient communities under the experimental conditions tested. This result mirrors the limited taxonomic convergence observed in the microbiota analyses and suggests that functional predictions associated with long-term contaminant exposure are difficult to transfer through short-term microbial exchange. However, we cannot exclude that the transplantation of a few taxa carrying these functions, or the indirect reshaping of the recipient microbiota following transplantation, may facilitate the increase of such taxa during subsequent chemical exposure, thereby enhancing the host response. In support of this possibility, recent microbiota transplantation experiments in bivalves suggested that donor-associated microbial communities may modulate host performance under acute thermal stress, leading to improved host transcriptional outcomes when recipients are challenged [30]. Moreover, these effects emerged only after exposure to a strong environmental stressor, suggesting that microbiome-mediated functional differences may become evident primarily under selective conditions rather than being expressed continuously.

In this context, the lack of enrichment of xenobiotic-related pathways under ‘not-contaminated-seawater’ conditions should not be read as evidence against their potential relevance. Rather, it reflects the fact that this study was designed to test the feasibility of microbiota transfer in the absence of chemical stress, while the functional consequences of donor-derived traits may become detectable under a targeted contamination challenge.

In addition, more accurate functional profiling is needed with future work that should combine predictive tools with metagenomic and metatranscriptomic pipelines [47]. The integration of these multi-omics datasets would enable the validation of predicted detoxification pathways and clarify whether donor-derived functions are transcriptionally active in recipient hosts.

### Concluding remarks

This study compared the potential and limitations of two microbiota transplantation approaches in adult bivalves. While both homogenate inoculation and cohabitation induced measurable changes in recipient prokaryotic community, these shifts remained limited in magnitude and transient over time, indicating strong host- and environment-driven constraints on donor establishment. The persistence of high community dissimilarity to the donor baseline, together with consistently low incorporation of donor-exclusive ASVs, is consistent with the ecological constraints on the integration of exogenous microorganisms into the digestive gland microbiome. Differential abundance analyses further indicated that most transplantation-associated community shifts involved taxa that were not characteristic of the donor community, suggesting general community restructuring rather than donor-specific microbiota acquisition.

Notably, ATB pretreatment, as applied in this study following the protocol described by Gallo *et al*. [28], did not achieve substantial depletion of the resident microbiota, as indicated by the lack of significant changes in both alpha diversity and total bacterial load, and did not enhance donor associated signal or donor-specific ASV acquisition. The incomplete perturbation of the resident microbiota therefore represents an important experimental limitation that may have contributed to the low transplantation efficiency. The results are also consistent with a potential role of host-associated ecological filtering, but the relative contributions of these factors cannot be resolved by the present experiment.

Future studies should therefore investigate how transplantation strategy, duration of donor exposure, and recipient ecological filtering interact to determine stable donor establishment.

Similarly, functional predictions revealed that xenobiotic-metabolism pathways predicted to be enriched in donor clams were not detectably acquired by recipient prokaryotic communities under short-term, non-selective conditions. This finding does not exclude a potential functional role of donor-derived taxa but suggests that microbiome-mediated functional differences are likely to become detectable only under appropriate environmental or physiological selective pressures.

Microbial communities may require longer periods to reach equilibrium, and later stages are more strongly influenced by environmental factors and tend to show reduced variability [48].

Accordingly, these findings outline both the potential and current limitations of microbiota manipulation in adult bivalves, providing an experimental framework for developing future transplantation strategies to address the ecological and experimental constraints identified in the present study. Such strategies may require more sustained or repeated microbial exposures, the targeting of early-life stages, and functional validation under relevant environmental stress conditions to assess the persistence and impact of donor-derived traits.

Taken together, these findings indicate that successful microbiota transplantation in adult bivalves will require not only improved strategies for recipient microbiota depletion, but also a better understanding of the host-associated ecological filtering that constrains donor establishment.

## Supplementary Material

Supplementary_material_ycag271

## Data Availability

The raw 16S rRNA gene sequencing data generated during this study are available in the NCBI Sequence Read Archive (SRA) under BioProject accession PRJNA1362988.
