# Supplementary material for: To cohabit or inoculate? Testing the limits of microbiota transplantation in Manila clams
Source: ISME Commun. 2026 Sep 25;6(1):ycag271. doi: 10.1093/ismeco/ycag271 (PMC13653611; doi:10.1093/ismeco/ycag271)
Supplement: Supplementary file 1 [file supplementary_material_ycag271.zip › Supplementary_Figure_S1_ycag271.pdf]

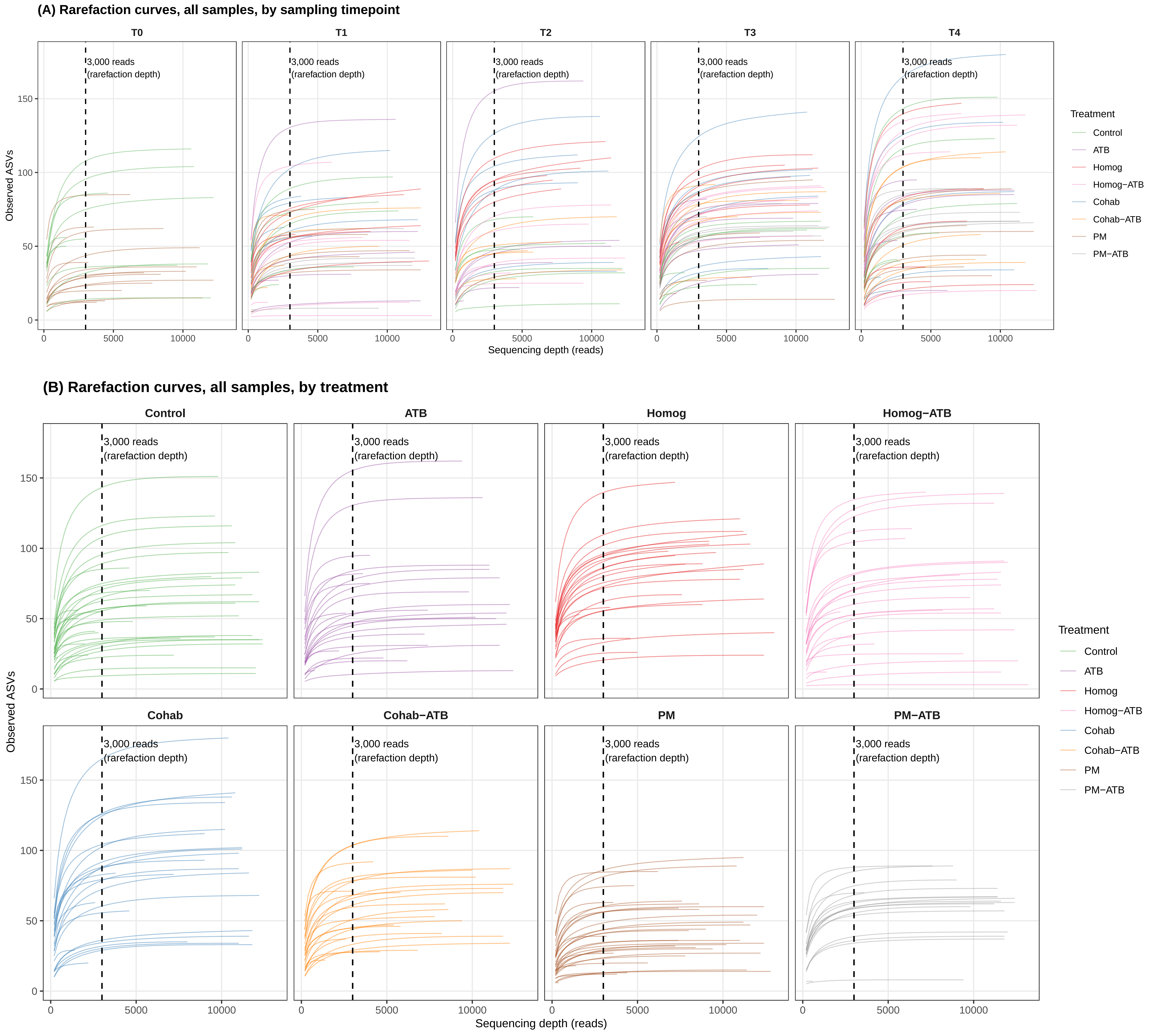
(A) Rarefaction curves, all samples, by sampling timepoint
T0
T1
T2
T3
T4
3,000 reads
(rarefaction depth)
Observed ASVs
Sequencing depth (reads)
Treatment
Control
ATB
Homog
Homog−ATB
Cohab
Cohab−ATB
PM
PM−ATB
(B) Rarefaction curves, all samples, by treatment
Control
ATB
Homog
Homog−ATB
Cohab
Cohab−ATB
PM
PM−ATB
3,000 reads
(rarefaction depth)
Observed ASVs
Sequencing depth (reads)
Treatment
Control
ATB
Homog
Homog−ATB
Cohab
Cohab−ATB
PM
PM−ATB
