# Supplementary material for: To cohabit or inoculate? Testing the limits of microbiota transplantation in Manila clams
Source: ISME Commun. 2026 Sep 25;6(1):ycag271. doi: 10.1093/ismeco/ycag271 (PMC13653611; doi:10.1093/ismeco/ycag271)
Supplement: Supplementary file 1 [file supplementary_material_ycag271.zip › Supplementary_Figure_S2_ycag271.pdf]

(A) DESeq2 fold change, T0, PM vs. Control (donor baseline)

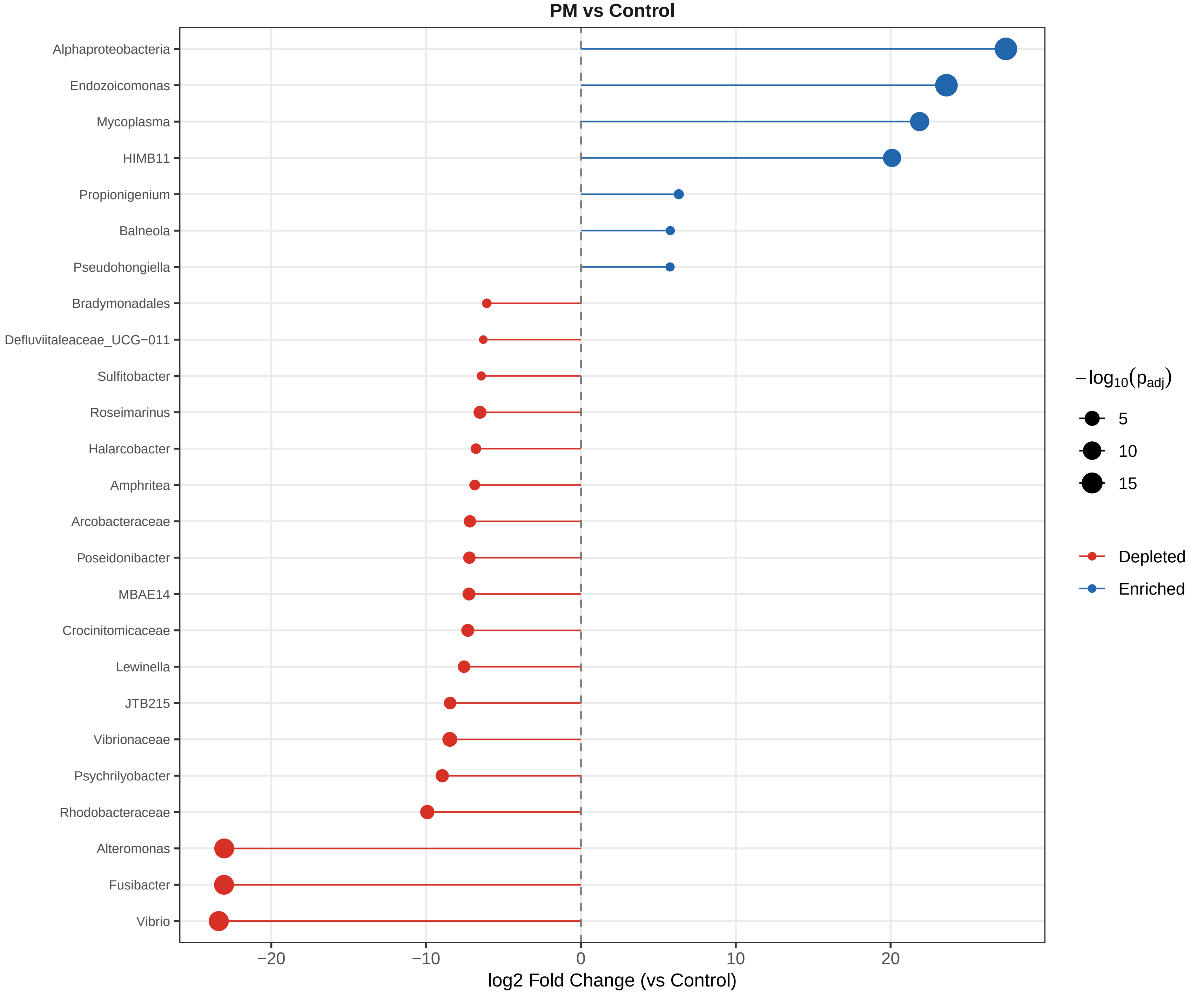

padj < 0.05 | |log2FC| > 1 | Blue = enriched vs Control | Red = depleted

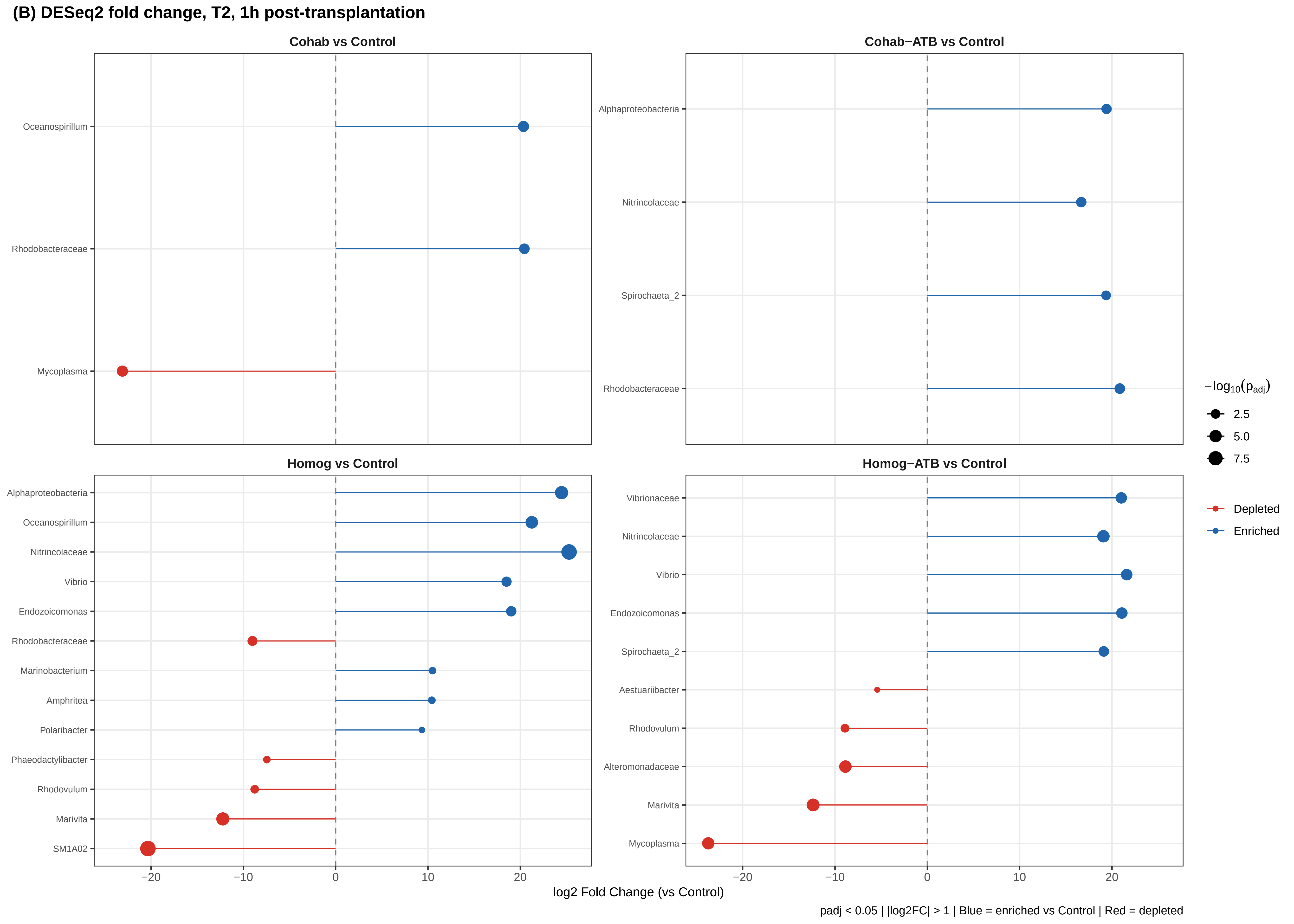
(B) DESeq2 fold change, T2, 1h post-transplantation
Cohab vs Control
Oceanospirillum
Rhodobacteraceae
Mycoplasma
Cohab−ATB vs Control
Alphaproteobacteria
Nitrincolaceae
Spirochaeta_2
Rhodobacteraceae
Homog vs Control
Alphaproteobacteria
Oceanospirillum
Nitrincolaceae
Vibrio
Endozoicomonas
Rhodobacteraceae
Marinobacterium
Amphritea
Polaribacter
Phaeodactylibacter
Rhodovulum
Marivita
SM1A02
Homog−ATB vs Control
Vibrionaceae
Nitrincolaceae
Vibrio
Endozoicomonas
Spirochaeta_2
Aestuariibacter
Rhodovulum
Alteromonadaceae
Marivita
Mycoplasma
−20
−10
0
10
20
log2 Fold Change (vs Control)
$-\log_{10}(p_{adj})$
2.5
5.0
7.5
Depleted
Enriched
padj < 0.05 | |log2FC| > 1 | Blue = enriched vs Control | Red = depleted

(C) DESeq2 fold change, T3, 3 days post-transplantation

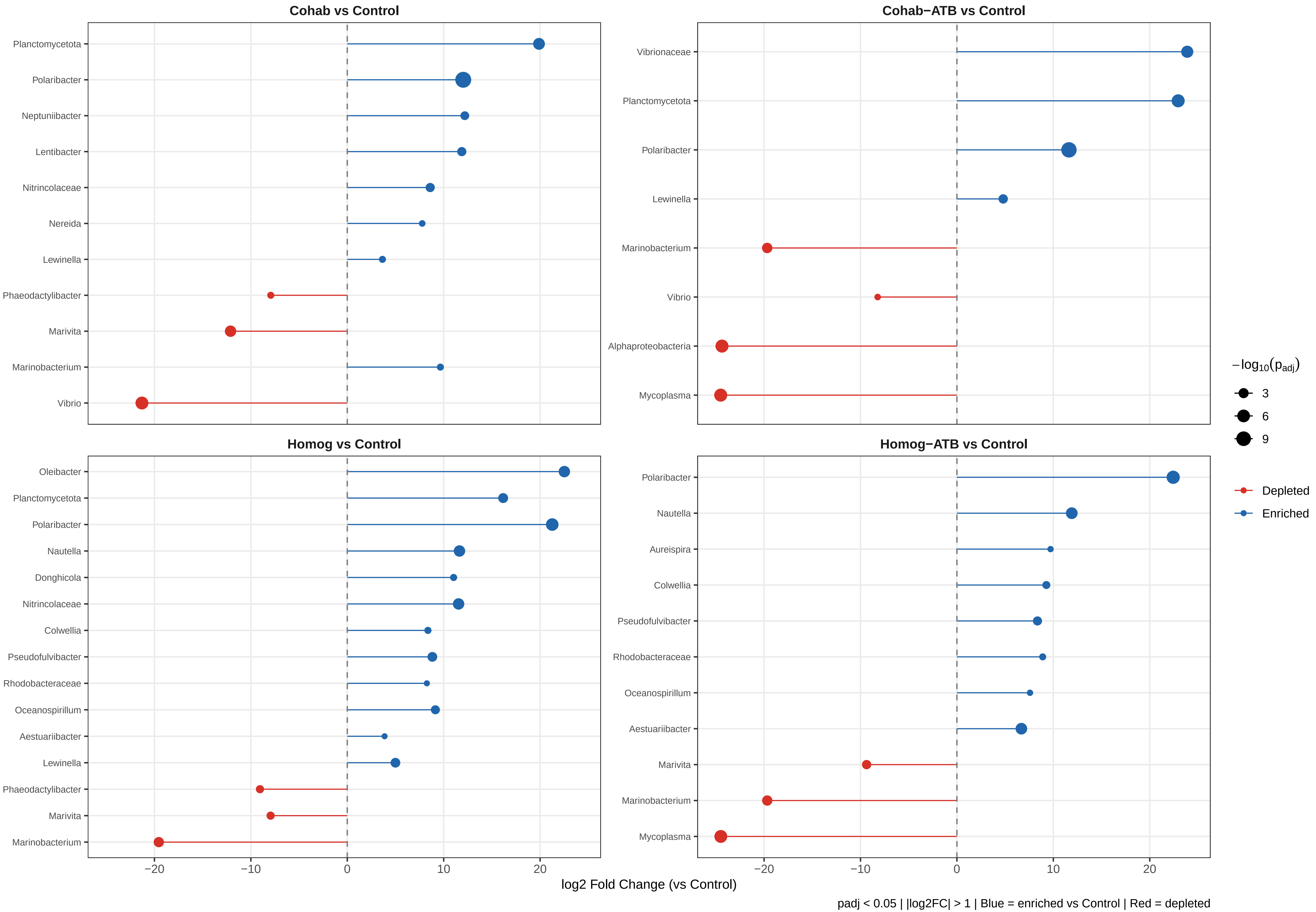

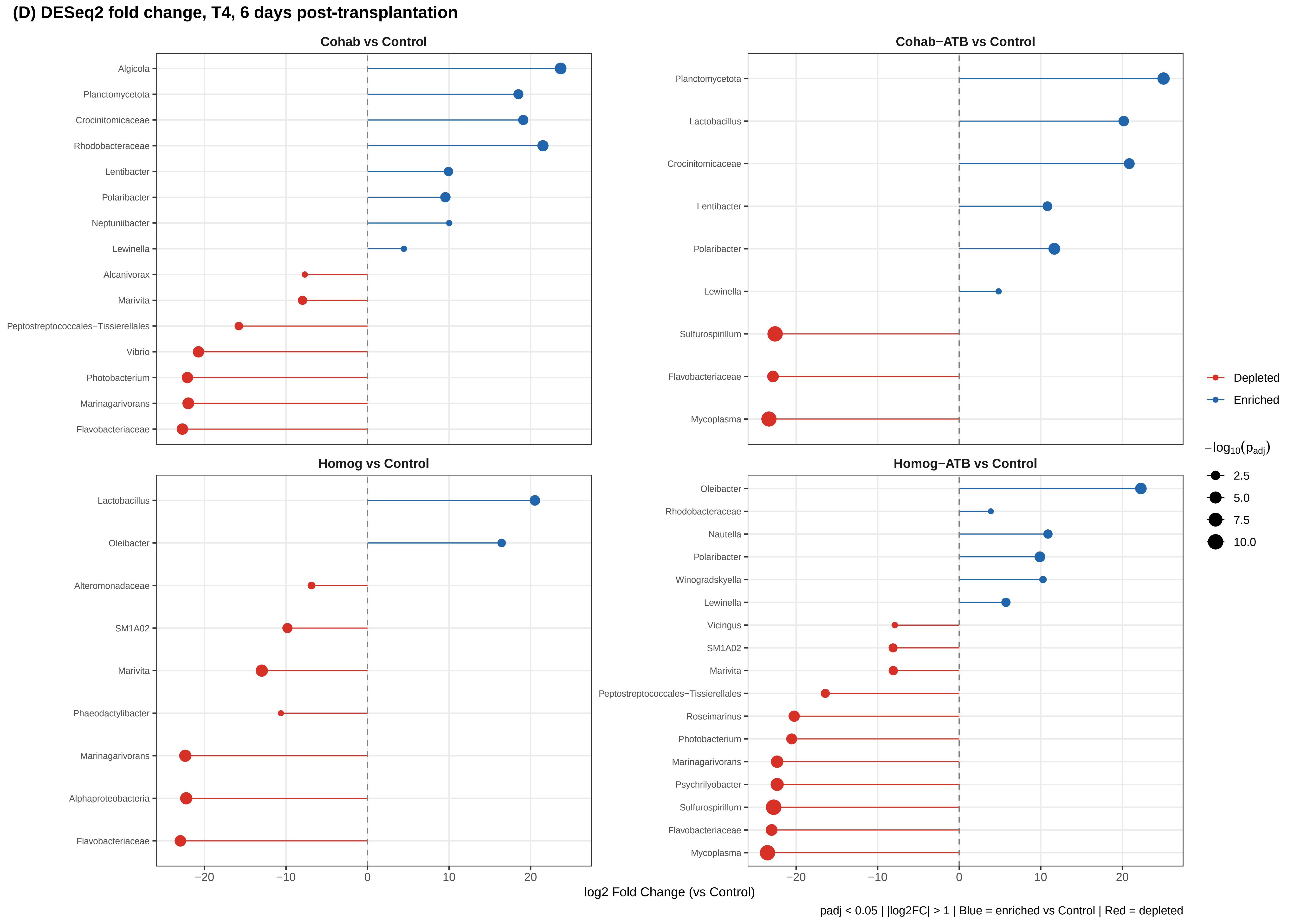

(D) DESeq2 fold change, T4, 6 days post-transplantation
Cohab vs Control
Algicola
Planctomycetota
Crocinitomicaceae
Rhodobacteraceae
Lentibacter
Polaribacter
Neptuniibacter
Lewinella
Alcanivorax
Marivita
Peptostreptococcales−Tissierellales
Vibrio
Photobacterium
Marinagarivorans
Flavobacteriaceae
Cohab−ATB vs Control
Planctomycetota
Lactobacillus
Crocinitomicaceae
Lentibacter
Polaribacter
Lewinella
Sulfurospirillum
Flavobacteriaceae
Mycoplasma
Homog vs Control
Lactobacillus
Oleibacter
Alteromonadaceae
SM1A02
Marivita
Phaeodactylibacter
Marinagarivorans
Alphaproteobacteria
Flavobacteriaceae
Homog−ATB vs Control
Oleibacter
Rhodobacteraceae
Nautella
Polaribacter
Winogradskyella
Lewinella
Vicingus
SM1A02
Marivita
Peptostreptococcales−Tissierellales
Roseimarinus
Photobacterium
Marinagarivorans
Psychrilyobacter
Sulfurospirillum
Flavobacteriaceae
Mycoplasma
−20
−10
0
10
20
log2 Fold Change (vs Control)
Depleted
Enriched
$-\log_{10}(p_{adj})$
2.5
5.0
7.5
10.0
padj < 0.05 | |log2FC| > 1 | Blue = enriched vs Control | Red = depleted
