# Supplementary material for: To cohabit or inoculate? Testing the limits of microbiota transplantation in Manila clams
Source: ISME Commun. 2026 Sep 25;6(1):ycag271. doi: 10.1093/ismeco/ycag271 (PMC13653611; doi:10.1093/ismeco/ycag271)

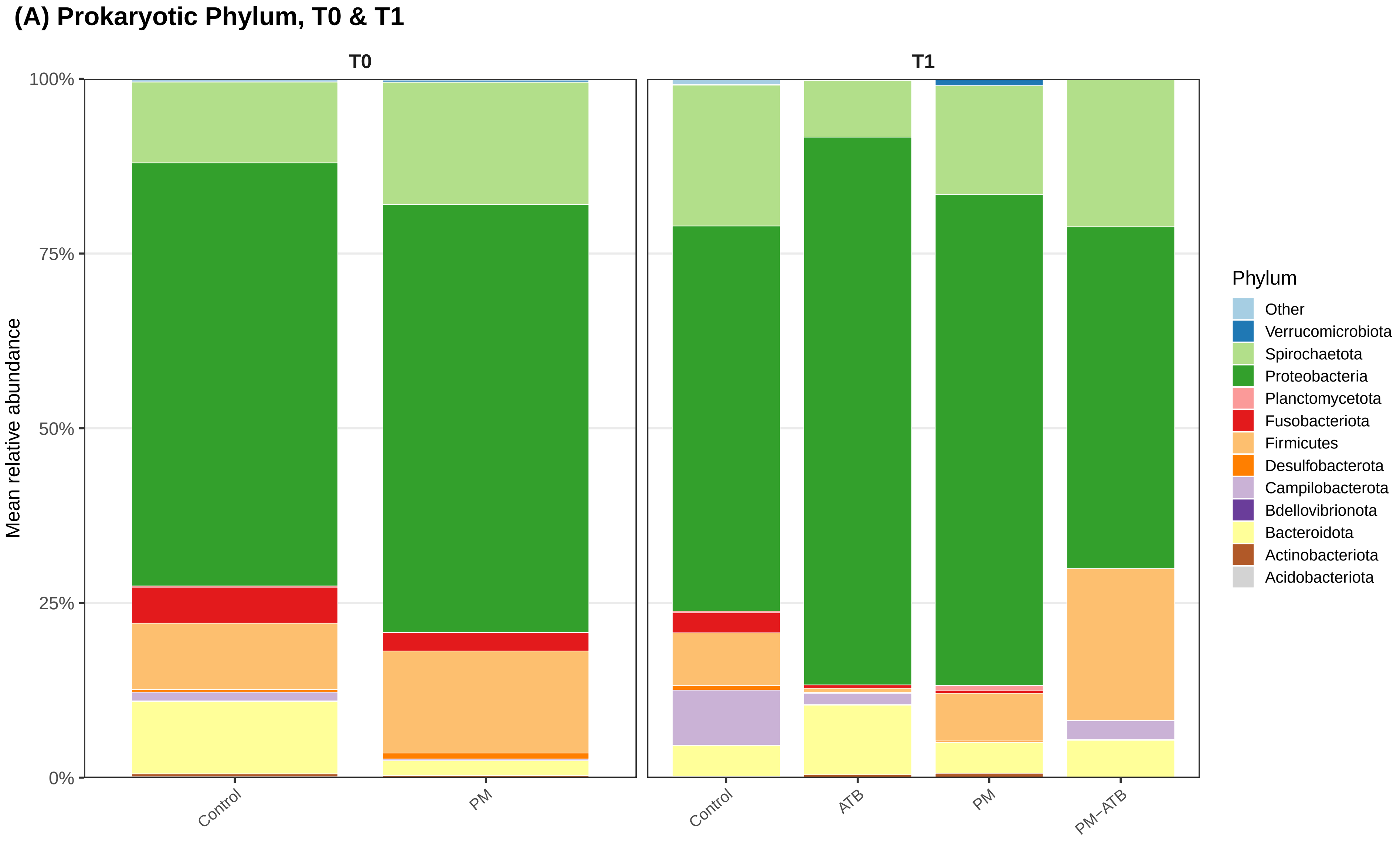

(A) Prokaryotic Phylum, T0 & T1
T0
T1
100%
75%
50%
25%
0%
Mean relative abundance
Control
PM
Control
ATB
PM
PM–ATB
Phylum
Other
Verrucomicrobiota
Spirochaetota
Proteobacteria
Planctomycetota
Fusobacteriota
Firmicutes
Desulfobacterota
Campilobacterota
Bdellovibrionota
Bacteroidota
Actinobacteriota
Acidobacteriota

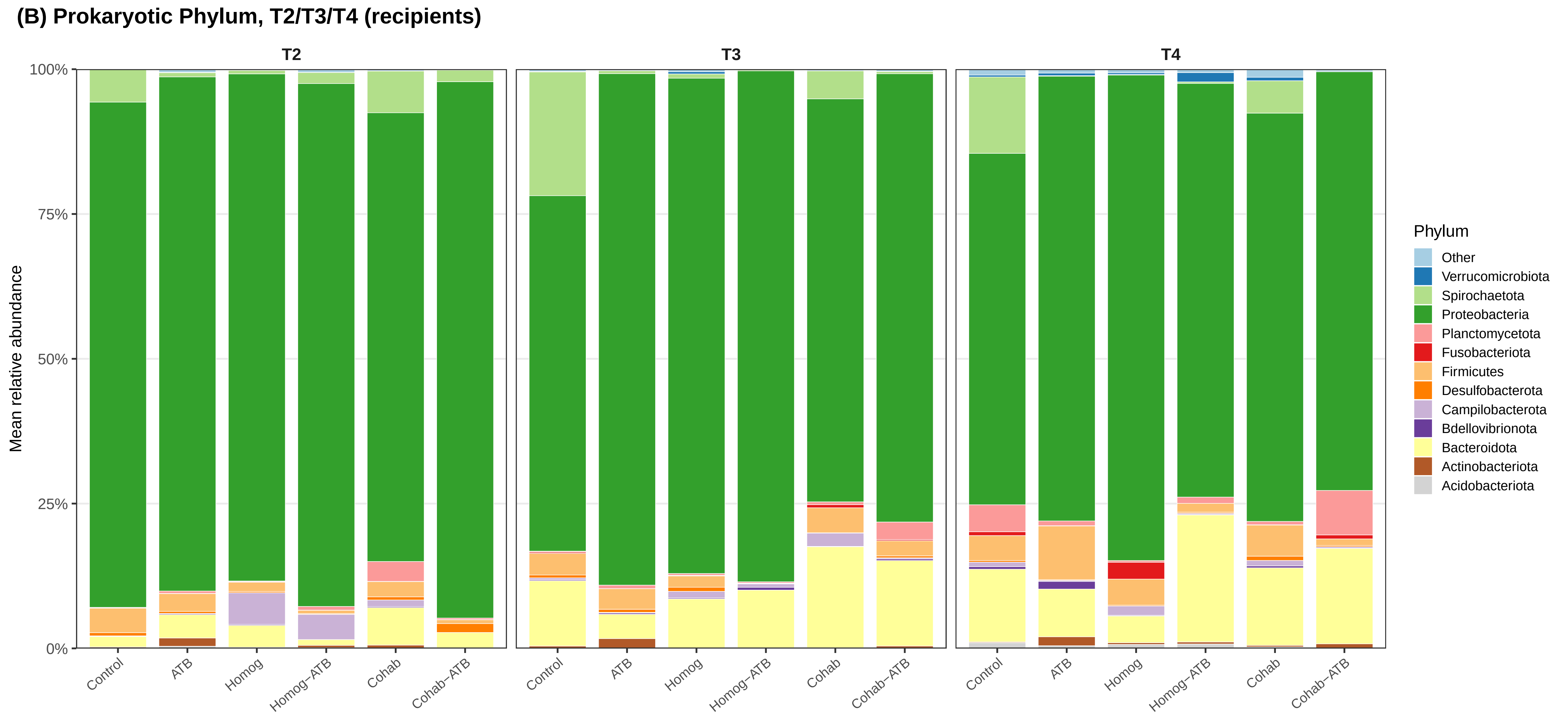
(B) Prokaryotic Phylum, T2/T3/T4 (recipients)
T2
T3
T4
Mean relative abundance
100%
75%
50%
25%
0%
Control
ATB
Homog
Homog−ATB
Cohab
Cohab−ATB
Phylum
Other
Verrucomicrobiota
Spirochaetota
Proteobacteria
Planctomycetota
Fusobacteriota
Firmicutes
Desulfobacterota
Campilobacterota
Bdellovibrionota
Bacteroidota
Actinobacteriota
Acidobacteriota

(C) Prokaryotic Phylum, T2/T3/T4 with PM T0 reference

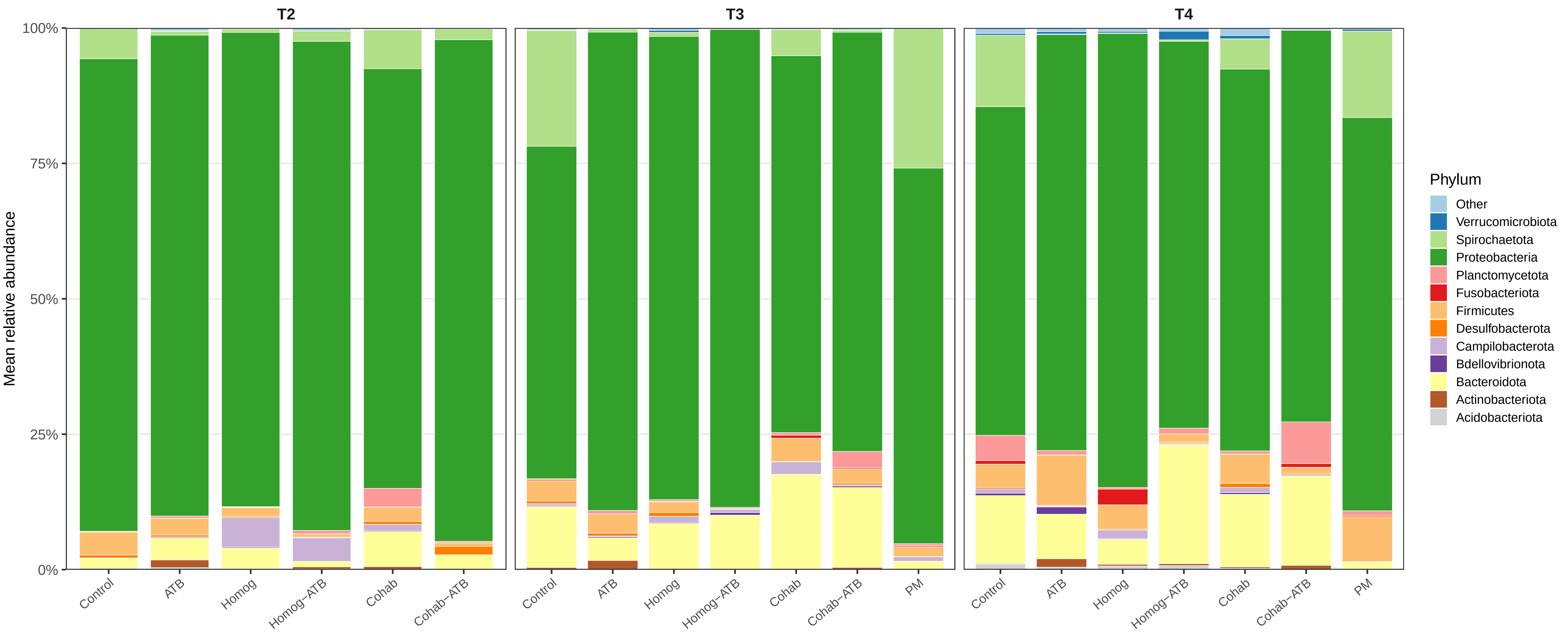

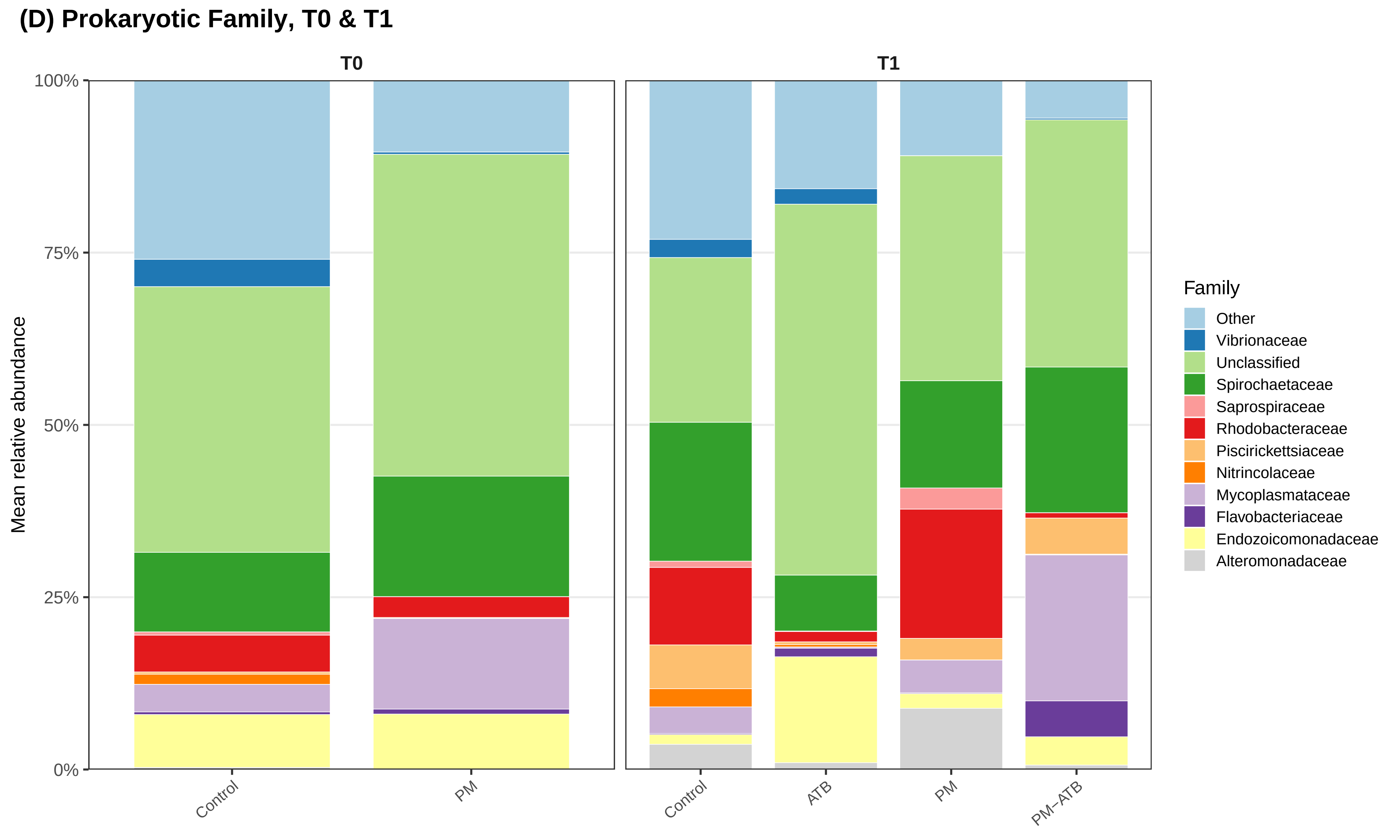

(D) Prokaryotic Family, T0 & T1
T0
T1
100%
75%
50%
25%
0%
Mean relative abundance
Control
PM
Control
ATB
PM
PM−ATB
Family
Other
Vibrionaceae
Unclassified
Spirochaetaceae
Saprospiraceae
Rhodobacteraceae
Piscirickettsiaceae
Nitrincolaceae
Mycoplasmataceae
Flavobacteriaceae
Endozoicomonadaceae
Alteromonadaceae

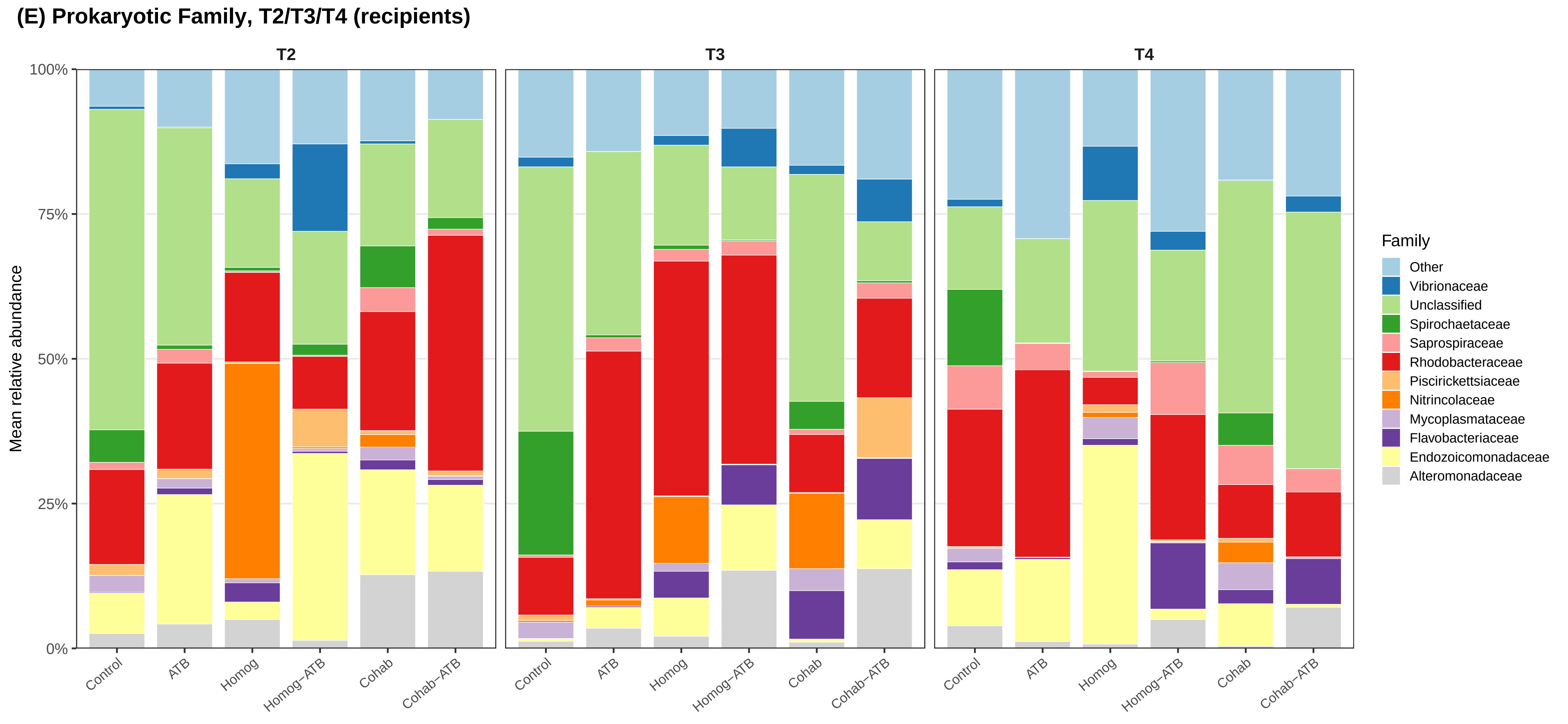
(E) Prokaryotic Family, T2/T3/T4 (recipients)
T2
T3
T4
Mean relative abundance
100%
75%
50%
25%
0%
Control
ATB
Homog
Homog−ATB
Cohab
Cohab−ATB
Family
Other
Vibrionaceae
Unclassified
Spirochaetaceae
Saprospiraceae
Rhodobacteraceae
Piscirickettsiaceae
Nitrincolaceae
Mycoplasmataceae
Flavobacteriaceae
Endozoicomonadaceae
Alteromonadaceae

(F) Prokaryotic Family, T2/T3/T4 with PM T0 reference

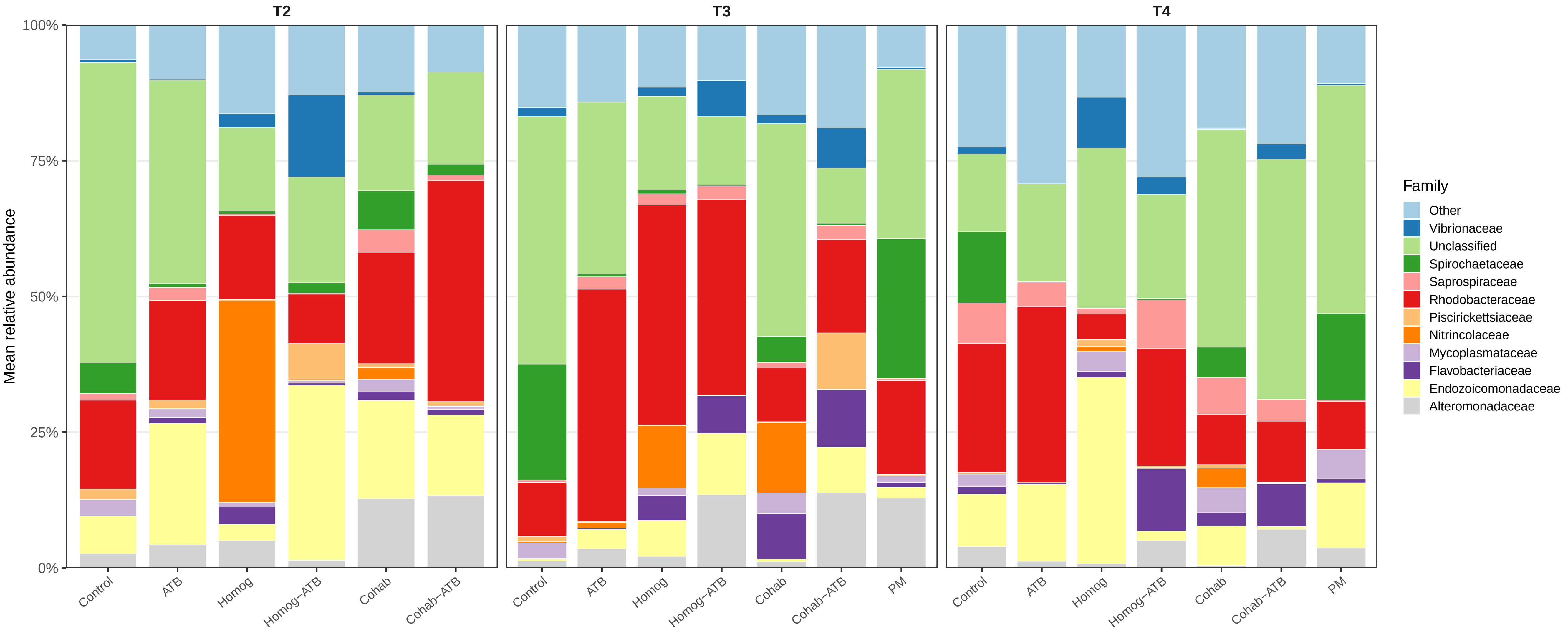

(G) Prokaryotic Genus, T0 & T1

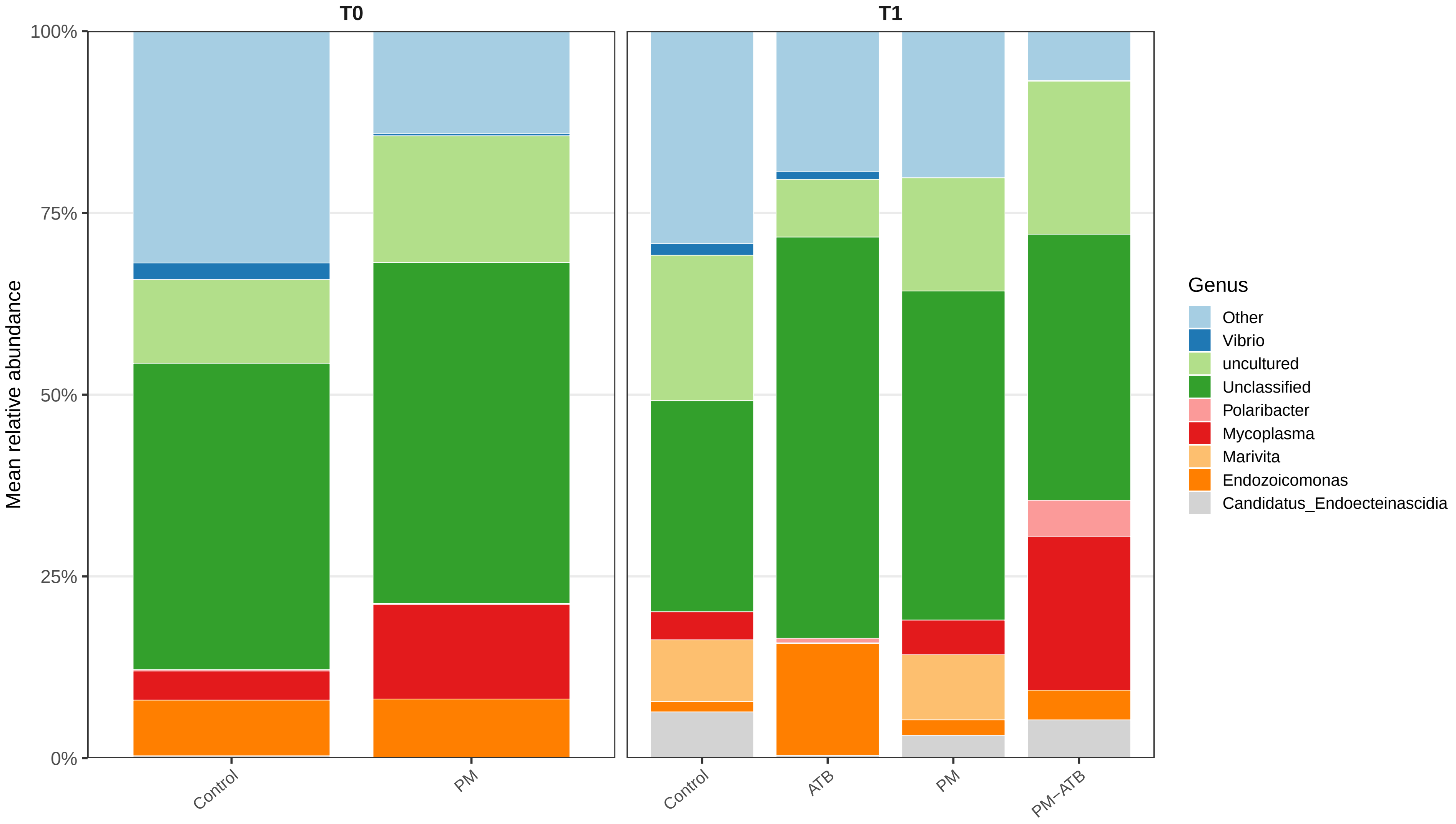

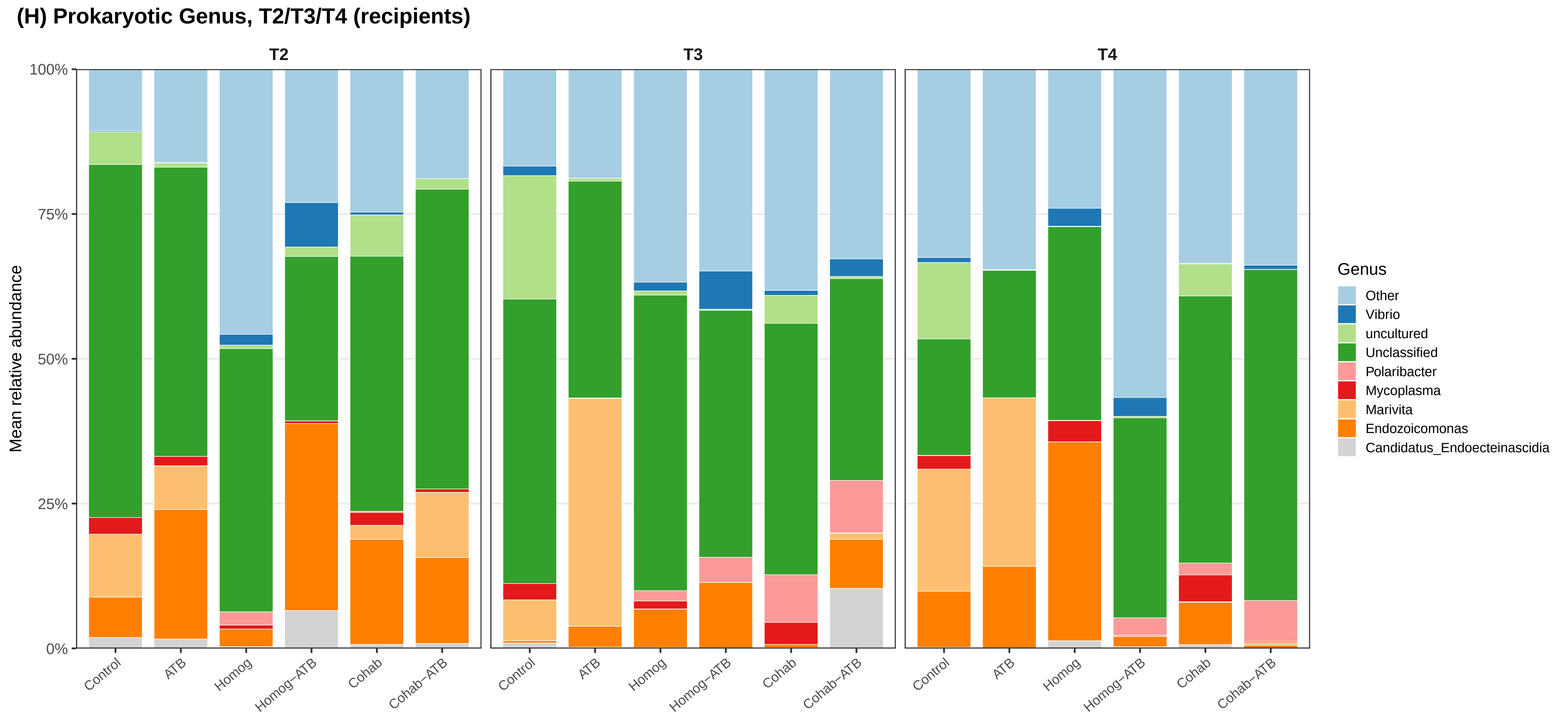
(H) Prokaryotic Genus, T2/T3/T4 (recipients)
T2
T3
T4
Mean relative abundance
100%
75%
50%
25%
0%
Control
ATB
Homog
Homog−ATB
Cohab
Cohab−ATB
Genus
Other
Vibrio
uncultured
Unclassified
Polaribacter
Mycoplasma
Marivita
Endozoicomonas
Candidatus_Endoecteinascidia

(I) Prokaryotic Genus, T2/T3/T4 with PM T0 reference

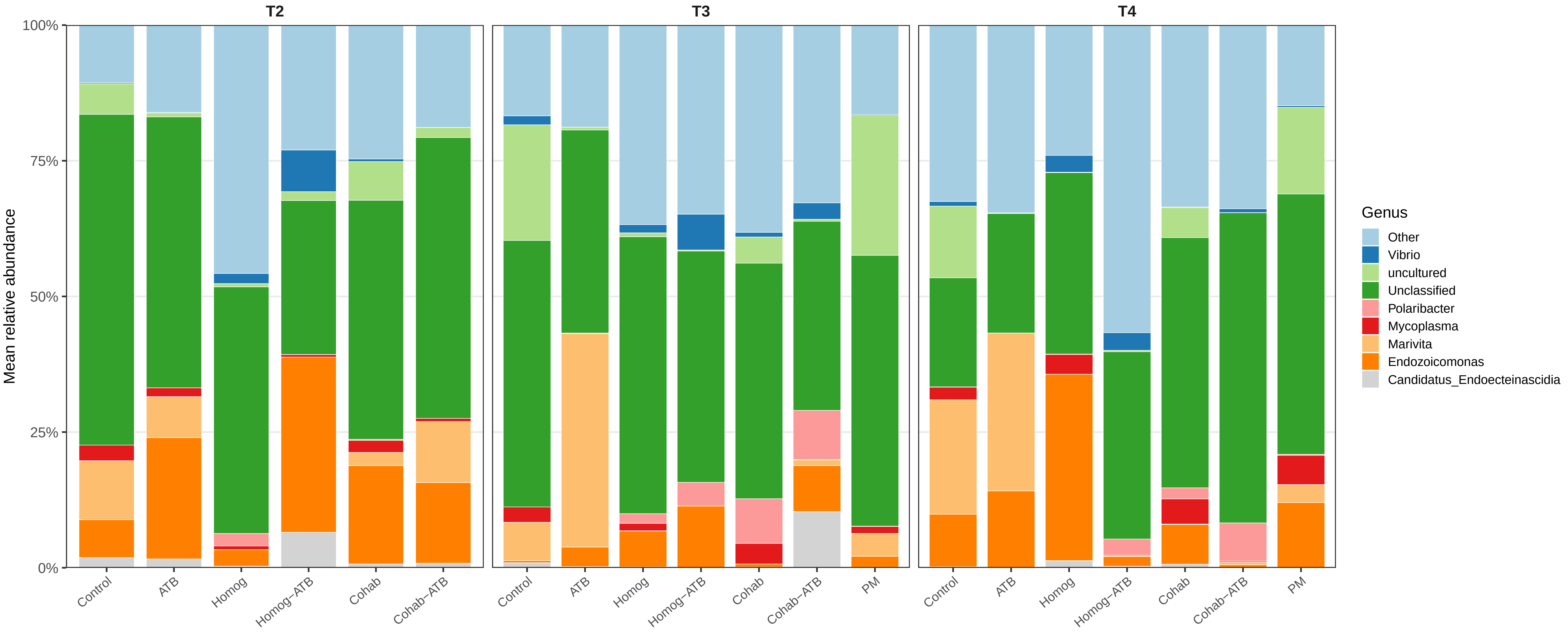

Supplement: Supplementary file 1 [file supplementary_material_ycag271.zip › Supplementary_Figure_S3_ycag271.pdf]
