# Supplementary material for: To cohabit or inoculate? Testing the limits of microbiota transplantation in Manila clams
Source: ISME Commun. 2026 Sep 25;6(1):ycag271. doi: 10.1093/ismeco/ycag271 (PMC13653611; doi:10.1093/ismeco/ycag271)

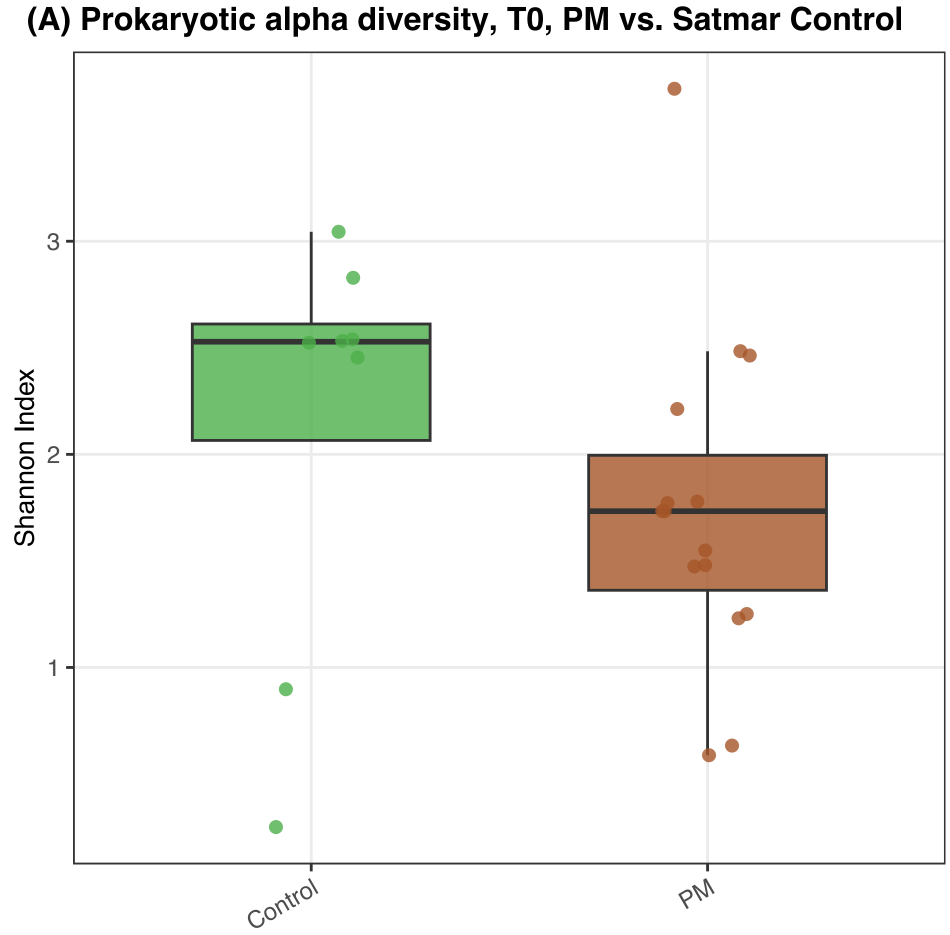
(A) Prokaryotic alpha diversity, T0, PM vs. Satmar Control
Shannon Index
3
2
1
Control
PM

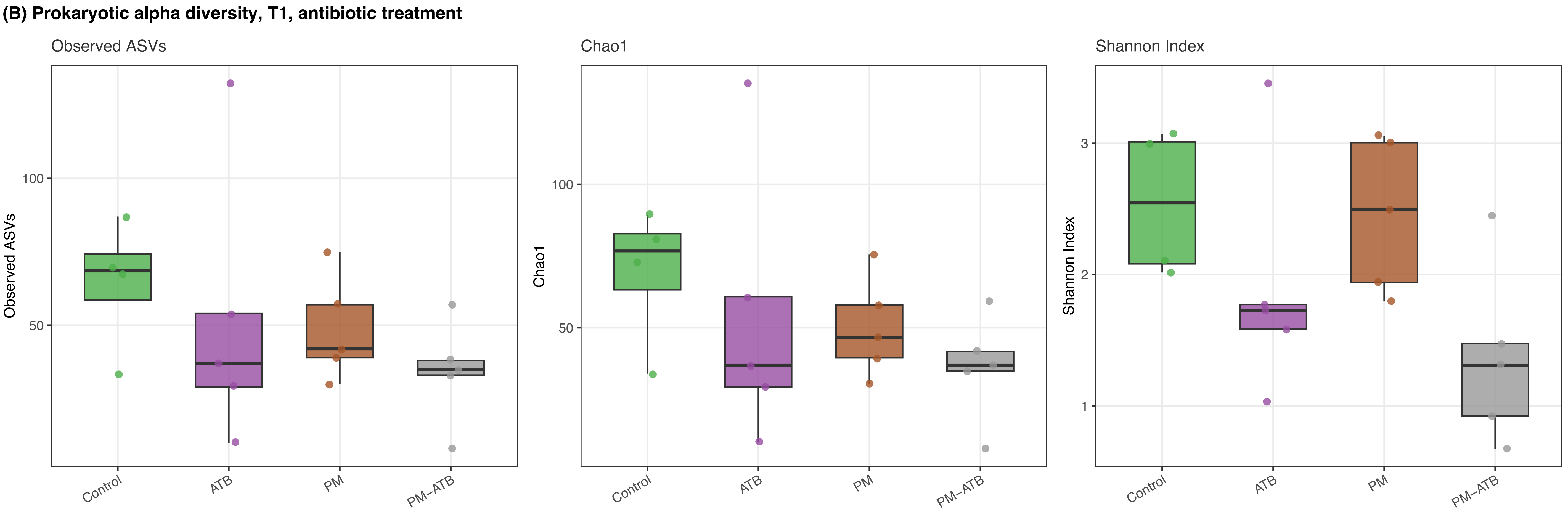
(B) Prokaryotic alpha diversity, T1, antibiotic treatment
Observed ASVs
Chao1
Shannon Index
Observed ASVs
100
50
Chao1
100
50
Shannon Index
3
2
1
Control
ATB
PM
PM–ATB

(C) Prokaryotic alpha diversity, T2, 1h post-transplantation

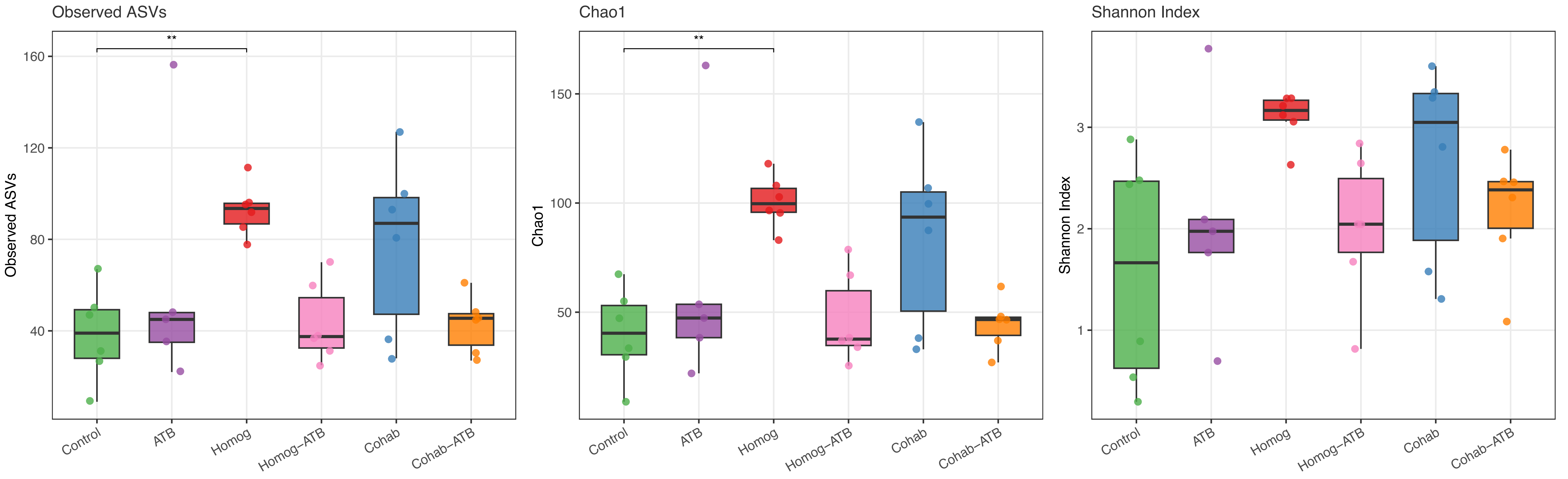

(D) Prokaryotic alpha diversity, T3, 3 days post-transplantation

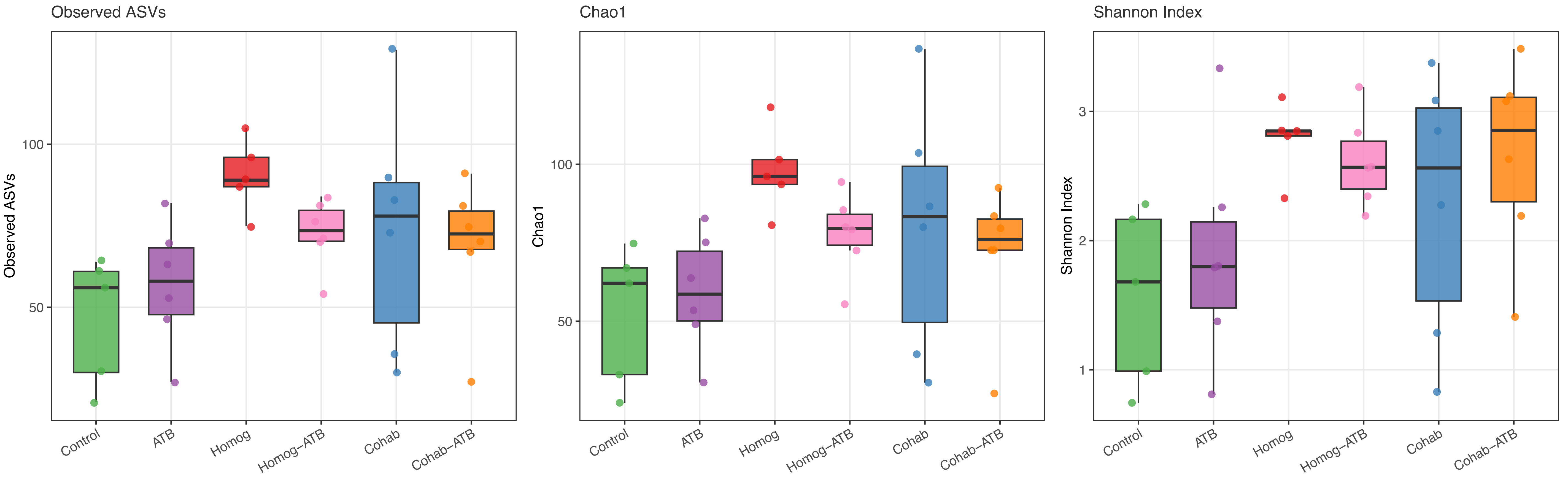

(E) Prokaryotic alpha diversity, T4, 6 days post-transplantation

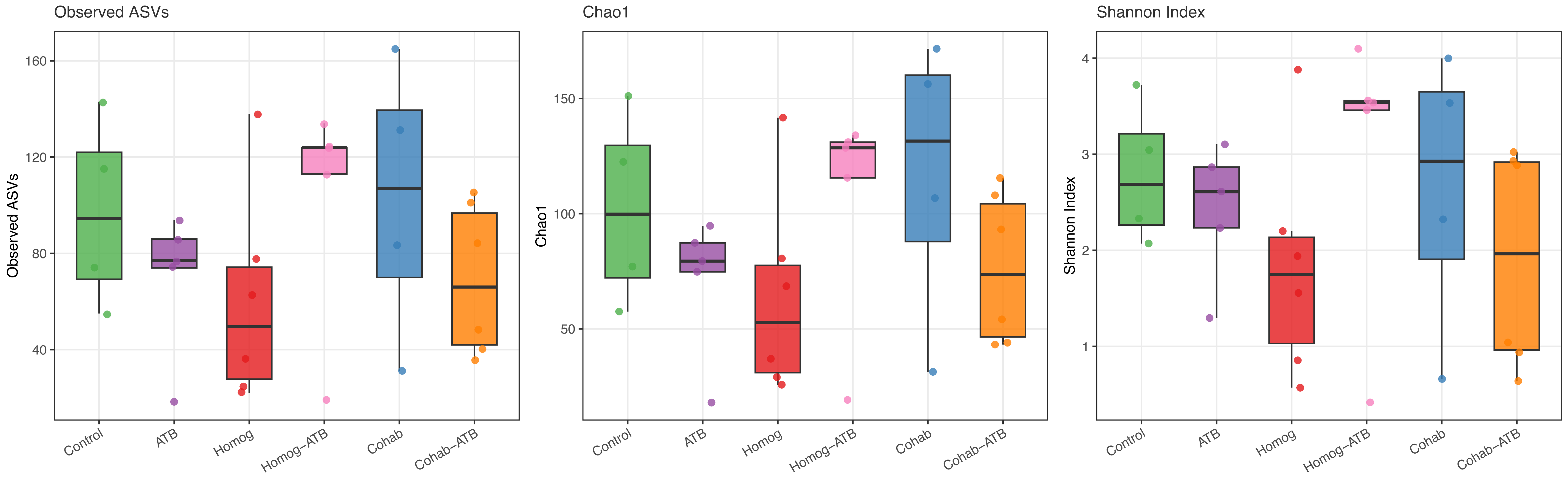

Supplement: Supplementary file 1 [file supplementary_material_ycag271.zip › Supplementary_Figure_S4_ycag271.pdf]
