# Supplementary material for: To cohabit or inoculate? Testing the limits of microbiota transplantation in Manila clams
Source: ISME Commun. 2026 Sep 25;6(1):ycag271. doi: 10.1093/ismeco/ycag271 (PMC13653611; doi:10.1093/ismeco/ycag271)
Supplement: Supplementary file 1 [file supplementary_material_ycag271.zip › Supplementary_Figure_S5_ycag271.pdf]

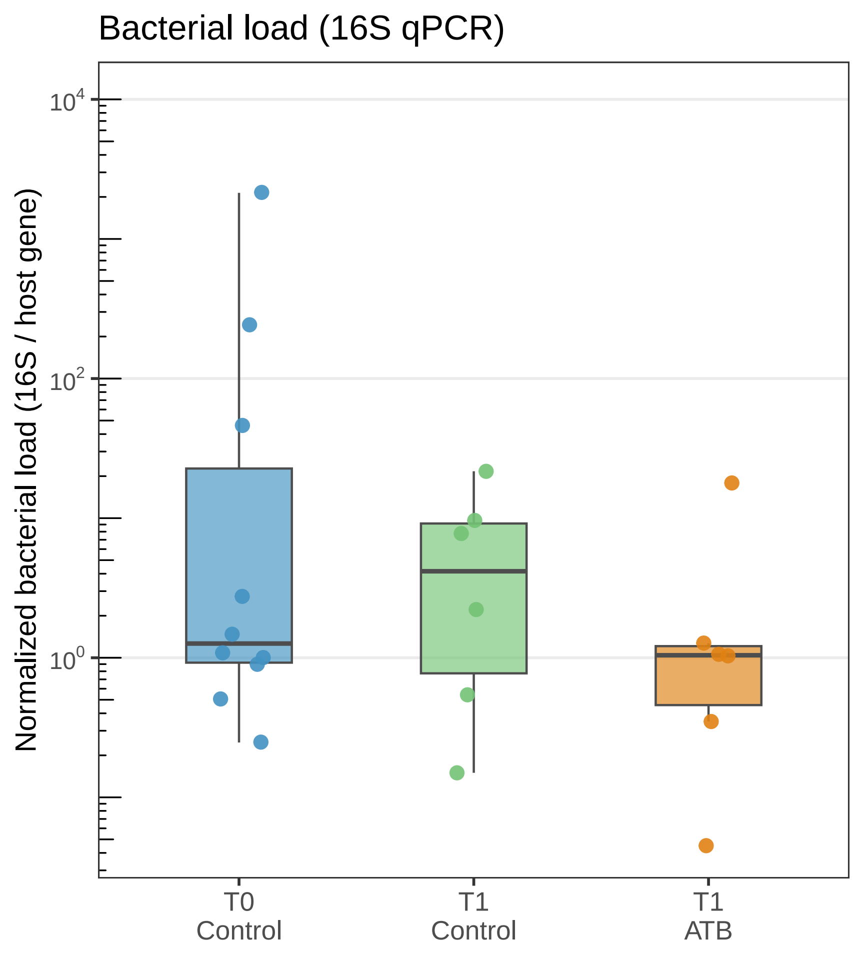

Bacterial load (16S qPCR)
Normalized bacterial load (16S / host gene)
10^4
10^2
10^0
T0
Control
T1
Control
T1
ATB
