# Supplementary material for: To cohabit or inoculate? Testing the limits of microbiota transplantation in Manila clams
Source: ISME Commun. 2026 Sep 25;6(1):ycag271. doi: 10.1093/ismeco/ycag271 (PMC13653611; doi:10.1093/ismeco/ycag271)
Supplement: Supplementary file 1 [file supplementary_material_ycag271.zip › Supplementary_Figure_S6_ycag271.pdf]

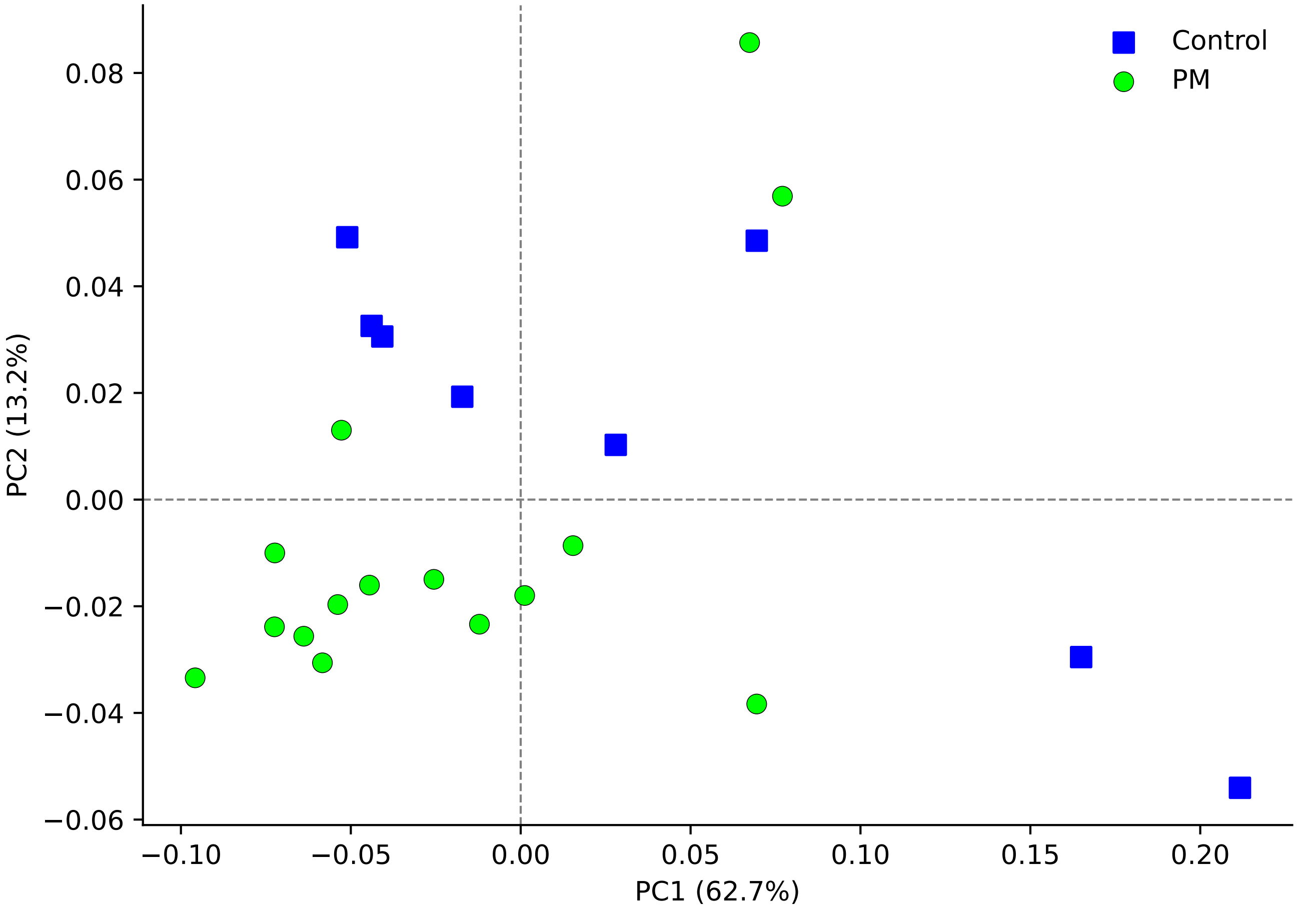

Control
PM
0.08
0.06
0.04
0.02
0.00
−0.02
−0.04
−0.06
PC2 (13.2%)
−0.10
−0.05
0.00
0.05
0.10
0.15
0.20
PC1 (62.7%)
