# Supplementary material for: To cohabit or inoculate? Testing the limits of microbiota transplantation in Manila clams
Source: ISME Commun. 2026 Sep 25;6(1):ycag271. doi: 10.1093/ismeco/ycag271 (PMC13653611; doi:10.1093/ismeco/ycag271)
Supplement: Supplementary file 1 [file supplementary_material_ycag271.zip › Supplementary_S1_final_ycag271.docx]

Supplementary Table S1. Summary of ASV filtering and sequencing depth.

| Category | Parameter | Value |
| --- | --- | --- |
| Filtering | ASVs removed (unclassified / mitochondria / chloroplast) | 558 |
| Filtering | ASVs removed (present in < 2 samples; prevalence filter) | 8945 |
| Final dataset | Total ASVs retained | 1570 |
| Sequencing depth | Minimum reads per sample | 601 |
| Sequencing depth | Median reads per sample | 10368 |
| Sequencing depth | Mean reads per sample | 9002 ± 3505 (mean ± SD) |
| Sequencing depth | Maximum reads per sample | 13240 |
| Rarefaction | Rarefaction depth used | 3,000 reads/sample |
| Rarefaction | Samples retained after rarefaction | 184 of 205 |

*Note: sequencing-depth values (minimum, median, mean ± SD, maximum) are reported for the final quality-filtered dataset (n = 205 samples); the rarefaction rows report the outcome of rarefying to 3,000 reads/sample, as described in the Methods.*
